# Aqueous-phase photo-oxidation of selected green leaf volatiles initiated by ^•^OH radicals: Products and atmospheric implications

**DOI:** 10.1016/j.scitotenv.2023.162622

**Published:** 2023-06-25

**Authors:** Kumar Sarang, Tobias Otto, Sahir Gagan, Krzysztof Rudzinski, Thomas Schaefer, Martin Brüggemann, Irena Grgić, Adam Kubas, Hartmut Herrmann, Rafal Szmigielski

**Affiliations:** a Institute of Physical Chemistry Polish Academy of Sciences, 01-224 Warsaw, Poland; b Atmospheric Chemistry Department (ACD), Leibniz Institute for Tropospheric Research (TROPOS), 04318 Leipzig, Germany; c Department of Analytical Chemistry, National Institute of Chemistry, SI-1000 Ljubljana, Slovenia

**Keywords:** Green leaf volatiles, Hydroxyl radicals, Aqueous phase, Reaction products, Secondary organic aerosol, DFT calculations

## Abstract

C_5_- and C_6_- unsaturated oxygenated organic compounds emitted by plants under stress like cutting, freezing or drying, known as Green Leaf Volatiles (GLVs), may clear some of the existing uncertainties in secondary organic aerosol (SOA) budget. The transformations of GLVs are a potential source of SOA components through photo-oxidation processes occurring in the atmospheric aqueous phase. Here, we investigated the aqueous photo-oxidation products from three abundant GLVs (1-penten-3-ol, (*Z*)-2-hexen-1-ol, and (*E*)-2-hexen-1-al) induced by •OH radicals, carried out in a photo-reactor under simulated solar conditions. The aqueous reaction samples were analyzed using advanced hyphenated mass spectrometry techniques: capillary gas chromatography mass spectrometry (c-GC–MS); and reversed-phase liquid chromatography high resolution mass spectrometry (LC-HRMS). Using carbonyl-targeted c-GC–MS analysis, we confirmed the presence of propionaldehyde, butyraldehyde, 1-penten-3-one, and 2-hexen-1-al in the reaction samples. The LC-HRMS analysis confirmed the presence of a new carbonyl product with the molecular formula C_6_H_10_O_2_, which probably bears the hydroxyhexenal or hydroxyhexenone structure. Density functional theory (DFT)-based quantum calculations were used to evaluate the experimental data and obtain insight into the formation mechanism and structures of the identified oxidation products *via* the addition and hydrogen-abstraction pathways. DFT calculations highlighted the importance of the hydrogen abstraction pathway leading to the new product C_6_H_10_O_2_. Atmospheric relevance of the identified products was evaluated using a set of physical property data like Henry's law constant (HLC) and vapor pressure (VP). The unknown product of molecular formula C_6_H_10_O_2_ has higher HLC and lower VP than the parent GLV and thus has potential to remain in the aqueous phase leading to possible aqueous SOA formation. Other observed carbonyl products are likely first stage oxidation products and precursors of aged SOA.

## Highlights

• OH-initiated aqueous photo-oxidation of green leaf volatiles produces SOA carbonyls.
• Propionaldehyde, butyraldehyde, 1-penten-3-one, and (2)-hexen-1-al were identified.
• DFT calculations reveal importance of OH-addition and H-abstraction mechanisms.
• Through precipitation, detected carbonyls affect all environmental spheres.

## Graphical abstract

**Figure:**
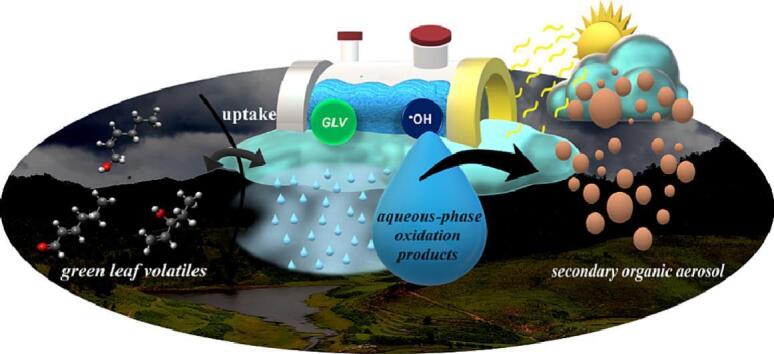

## 1 Introduction

Biogenic volatile organic compounds (BVOCs) emitted by terrestrial vegetation contribute largely to the formation of aerosols in the atmosphere (Kanakidou et al., 2005; Jimenez et al., 2009b), of which >70 % are secondary organic aerosols (SOA) (Kavouras et al., 1998; Kavouras and Stephanou, 2002; Hallquist et al., 2009; Jaoui et al., 2019; Iinuma et al., 2021). SOA are ubiquitous in the atmosphere (Pandis et al., 1992; Guenther et al., 1995; Claeys et al., 2004; Carlton et al., 2009; Khan et al., 2017). Understanding the role of atmospheric aerosols requires identifying and quantifying their sources and chemical composition (Kroll and Seinfeld, 2008; McNeill, 2015). Therefore, continuous development of knowledge about degradation processes and oxidation products formed during atmospheric reactions of BVOCs is essential to understand the impact of SOA on air quality (Karl et al., 2005; Stockwell et al., 2012), climate (Zhao et al., 2017), and human health (Particles, 2013).

Green leaf volatiles (GLVs) are a class of C_5_ – C_6_ unsaturated aldehydes, alcohols, or esters formed by the decomposition of C_18_-polyunsaturated fatty acids in leaves (*i.e.*, α-linolenic and linoleic acid) when a plant is exposed to stress due to extreme weather conditions (such as drought, high/low temperatures) or wounding (Fisher et al., 2003; Kleist et al., 2012; Blande et al., 2014; Matsui and Koeduka, 2016; Ameye et al., 2018). Examples of GLVs include: (*E*)-2-hexen-1-al, (*Z*)-3-hexenal, (*Z*)-2-hexen-1-ol, 1-penten-3-ol, and (*Z*)-3-hexen-1-ol (Kirstine et al., 1998; de Gouw et al., 1999; Kirstine and Galbally, 2004; Karl et al., 2005; Brilli et al., 2011; Jardine et al., 2012; Scala et al., 2013; Jardine et al., 2015). Based on a global estimation of SOA, a major part of the potential precursors is still unknown, and GLVs could be one of them (Goldstein and Galbally, 2007). More than half of global BVOCs emitted (1087 Tg yr^−1^) constitutes of isoprene (535 Tg yr^−1^) and monoterpenes (162 Tg yr^−1^), whereas the other 40–50 % is classified as other biogenic VOCs (390 Tg yr^−1^) (Vempati et al., 2018); they include GLVs, which can contribute significantly to the global SOA budget under specific conditions, such as plant stress (Guenther et al., 2012; Kleist et al., 2012).

Despite predictions that climate change will affect the vegetative volatile emissions and thus biogenic SOA formation, the SOA concentrations are underestimated in the models due to significant uncertainties in the knowledge of SOA precursors and mechanisms (Kanakidou et al., 2005; Fuzzi et al., 2006; Hallquist et al., 2009; McNeill, 2015; Shrivastava et al., 2017; Fan et al., 2022; Gao et al., 2022). Emissions and ambient concentrations of all GLVs are summarized in a review article (Sarang et al., 2021b). The global annual contribution of the most abundant GLVs (*Z*)-3-hexenal: 4.9 Tg yr^−1^, (*Z*)-3-hexenol: 2.9 Tg yr^−1^, and 2-methyl-3-buten-2-ol: 2.2 Tg yr^−1^ (Guenther et al., 2012) results in an estimated SOA of 0.58–1.05 Tg yr^−1^ (Sarang et al., 2021b) compared to the global annual output of 13–121 Tg yr^−1^ of SOA from all BVOCs (Tsigaridis et al., 2014). Local and seasonal variation in GLV emissions can be relatively large and contribute significantly to the air quality and SOA budget (Sarang et al., 2021b). A study conducted in Amazon tropical forests showed that emissions of C_6_ and C_5_ GLVs increased significantly during high temperatures in the afternoon, while emissions of volatile terpenoids (*e.g.*, isoprene, monoterpenes) decreased (Jardine et al., 2015). Published data on VOC emissions from the central Alps during the cool autumn of November 1999 (Karl et al., 2001) showed that C_6_ and C_5_ GLVs concentrations were 0.5–5 ppbv due to the freezing temperatures, which may be important for tropospheric chemistry.

Various studies using GLVs as SOA precursors have been conducted in the gas phase (Orlando et al., 2001; O'Connor et al., 2006; Hamilton et al., 2009; Jimenez et al., 2009a; O'Dwyer et al., 2010; Davis and Burkholder, 2011; Zhao et al., 2011; Gibilisco et al., 2013; Mentel et al., 2013; Shalamzari et al., 2014; Shalamzari et al., 2016) and at air-water interface (Liyana-Arachchi et al., 2013a; Liyana-Arachchi et al., 2013b; Liyana-Arachchi et al., 2013c; Liyana-Arachchi et al., 2014). The importance of aqueous-phase reactions between GLVs and atmospheric radicals has only recently been recognized, and a very few studies reported on their kinetics of the aqueous-phase oxidation and product formation (Richards-Henderson et al., 2014; Hansel et al., 2015; Richards-Henderson et al., 2015). Water droplets in clouds and fogs are diluted enough to dissolve the poorly soluble organic compounds (Facchini et al., 2000; McNeill, 2015; Pye et al., 2020), which can undergo photochemical or dark oxidation reactions and convert to less volatile compounds with a higher molecular weight, resulting in aqueous SOA (Andreae and Crutzen, 1997). Monod et al. (2005) also pointed out the importance of the aqueous droplets serving as a strong reacting medium for the removal of the unsaturated compounds from the atmosphere. Recently, we have described the aqueous-phase kinetics of 1-penten-3-ol (PENTOL), (*Z*)-2-hexen-1-ol (HEXOL), and (*E*)-2-hexen-1-al (HEXAL) with tropospheric radicals •OH, SO_4_•^-^ and NO_3_•, and evaluated their atmospheric significance (Sarang et al., 2021a). In the present study, we report on the chemical characterization of products obtained from aqueous photo-oxidation of the same three GLVs with •OH radicals, and discuss their possible role as SOA precursors. We used Xenon arc lamp as a light source to carry out photo-oxidation reaction in the photoreactor containing aqueous solution of the respective GLVs with a radical precursor. Hyphenated-mass spectrometry including a capillary gas chromatography–mass spectrometry (cGC-MS) and reversed-phase liquid chromatography high resolution mass spectrometry (LC-HRMS), was applied to characterize and quantify the organic products of the aqueous-phase reactions, while theoretical methods, such as density functional theory (DFT)-assisted quantum calculations, were used to validate the experimental findings.

HRMS techniques, such as LC-HRMS, provide an accurate elemental composition even at low atmospheric detection limits of concentration ranging from pg m^−3^ to ng m^−3^ while cGC-MS adds more structural information through detailed fragmentation analysis (Johnston and Kerecman, 2019). They are very popular in atmospheric research as they help to resolve the complex chemical composition of atmospheric matrices (Goldstein and Galbally, 2007). The details of the techniques and methods are discussed in the Section 2 and SI.

## 2 Material and methods

### 2.1 Materials

All chemicals were purchased and used without any purification: 1-penten-3-ol (Sigma Aldrich, 99.0 %), (*Z*)-2-hexen-1-ol (Sigma Aldrich, 95.0 %), (*E*)-2-hexen-1-al (Sigma Aldrich, 98.0 %), catalase from bovine liver (Sigma Aldrich, 10,000–40,000 units/mg protein), *o*-(2,3,4,5,6- pentafluorobenzyl) hydroxylamine hydrochloride (Sigma Aldrich, for GC derivatization, ≥ 99 % (AT)), hydrogen peroxide (H_2_O_2_, CHEMSOLUTE, 30.0 % wt. in H_2_O), acetonitrile (Optima, LC-MS grade), fuming hydrochloric acid (EMSURE, 37.0 %), 2,2,6,6-cyclohexanone-d_4_ (ISOTEC, 99 atom % D), 2,2,4-trimethyl pentane or isooctane (Merck, p.a. ≥ 99.5 % GC), formic acid LC-MS grade (Merck LiChropur, 98–100 % and Fisher Optima). Dichloromethane (Honeywell, ≥ 99.0 %), 1-penten-3-one (Sigma Aldrich, analytical standard with 0.1 % BHT as stabilizer), propionaldehyde (Merck, ≥ 98 %), butyraldehyde (Merck, ≥ 99 %), trans-2-hexenoic acid (Sigma Aldrich, 99 %). Aqueous solutions were freshly prepared using Milli-Q water (resistivity 18.2 MΏ cm, TOC < 3 ppb, Elix Millipore, Milli-Q gradient A10).

### 2.2 Experimental procedure

Details of the setup for product studies are similar to that used in previous study (Otto et al., 2019) and are also described and illustrated in SI Section 2.

Briefly, 300 mL of an aqueous solution containing a GLV (1 × 10^−4^ M) and hydrogen peroxide (5 × 10^−3^ M) was irradiated in the photo-reactor for 6 h (Fig. S1). Aliquots of reaction solution were taken every 15 min for the first 3 h and every 30 min later. Details of all experimental runs performed are described in Table S1. The reaction solution samples were collected in separate vials for capillary gas chromatography–mass spectrometry (cGC–MS) and reversed-phase liquid chromatography high resolution mass spectrometry (LC-HRMS) analyses. Carbonyl products were identified and quantified using a cGC–MS method (Rodigast et al., 2015) described in Section 2.2.1. The cGC–MS was also used for the analysis of GLVs and other alcohols (Section 2.2.2), while the LC-HRMS was used for quantification of GLVs, and for identification of other accretion products or carboxylic acids (Section 2.2.3). The method of product analysis is also described elsewhere (Otto et al., 2017, Otto et al., 2019). The GC–MS and LC-HRMS methods are included in SI, while the analysis procedure is briefly described here.

#### 2.2.1 Carbonyl-targeted GC–MS analyses

The 2 mL samples of the reaction solution were taken at specified intervals (see Section 2.2) to monitor the production of carbonyls during the experiments. A catalase solution (100 μL, γ = 5 mg mL^−1^) was added to each sample, followed by 100 μL of an internal standard 2,2,6,6-cyclohexanone-d_4_ (1.01 × 10^−4^ M), and finally 200 μL of the derivatizing agent O-(2,3,4,5,6-pentafluorobenzyl) hydroxylamine hydrochloride (γ = 5 mg mL^−1^). The samples were kept at 20 °C for at least 18 h for derivatization. Calibration standards for HEXAL, propionaldehyde (propanal), butyraldehyde (butanal), and 1-penten-3-one were prepared in the same way. Each sample was adjusted to pH = 1.0 ± 0.1 by addition of 60 μL hydrochloric acid (37 wt%), followed by extraction of the oxime derivatives by addition of 200 μL of 2,2,4-trimethylpentane or isooctane, and shaking for 30 min. An aliquot of 1 μL of the isooctane extracted organic layer containing derivatized carbonyl groups was injected in the cGC-MS system for analysis without prior drying.

#### 2.2.2 Alcohol-targeted GC-MS analyses

Reaction samples for alcohol-targeted GC-MS (this section) and LC-HRMS analyses (Section 2.2.3) were collected in a single LC vial. To avoid dark oxidation by excess H_2_O_2_, the catalase solution (25 μL, γ = 5 mg mL^−1^) was added (500 μL), followed by the addition of acetonitrile (500 μL), and stored at −25 °C until further analysis.

In the present case, GLVs (PENTOL and HEXOL) were quantified in the reaction sample and other alcohol products were identified by cGC-MS. A volume of 500 μL of the aliquot (1 mL) was transferred to another LC vial, 150 μL of dichloromethane (DCM) was added, and the solution was shaken in an orbital shaker for 30 min. The organic compounds containing alcohol functional group were extracted using DCM. Calibration standards of concentrations ranging from 1 × 10^−6^ to 1 × 10^−4^ M were prepared according to the same protocol. Since no internal standard was used in the present method, the calibration curve was obtained at the end of each sample run. The DCM extract (1 μL) was injected into the cGC-MS system without prior drying.

#### 2.2.3 LC-HRMS analyses

An ultra-high performance liquid chromatography system (UHPLC) coupled to a Q-Exactive PLUS Hybrid Quadrupole-Orbitrap mass spectrometer (UHPLC- PDA-HESI/MS) was used to analyze possible reaction products, such as carboxylic acids, of all three examined GLVs. Samples collected for alcohol-targeted GC-MS analyses were also used unchanged for LC-HRMS analyses. For quantification of HEXOL, LC-HRMS analysis with four separate sample injections, from each set of two individual experiments provided more reliable results than two injections in cGC-MS (no internal standard). While in the case of HEXAL, quantification using carbonyl-targeted cGC-MS analyses proved to be more accurate due to the use of both, internal and authentic standard. For quantification of HEXOL and HEXAL in the reaction samples, calibration standards ranging from 1 × 10^−6^ to 1 × 10^−4^ M were prepared using the same protocol as described above. Both photodiode array (PDA) and MS (ion chromatogram) detectors were used to quantify GLVs and their aqueous oxidation products.

### 2.3 Density functional theory calculations

Computational investigation with the density functional theory (DFT) was performed using the ORCA 5.0 suite of programs (Neese et al., 2020; Neese, 2022). The B3LYP hybrid functional (Vosko et al., 1980; Lee et al., 1988; Becke, 1993; Stephens et al., 1994) extended with the Grimme's dispersion correction, D3BJ (Grimme et al., 2010; Grimme et al., 2011) was used for the calculations. In all cases, def2-TZVP basis set (Weigend and Ahlrichs, 2005) was employed. The coulomb and exchange integrals were efficiently evaluated using the resolution-of-identity, RI (Eichkorn et al., 1995; Eichkorn et al., 1997) and chain-of-spheres, COSX (Izsak and Neese, 2011) approximations, respectively. The appropriate RI auxiliary basis set of Weigend (Weigend, 2006) was used throughout the study. All geometric parameters of the studied reactants, reaction complexes (denoted as RC), transition states (TS) and products (P—H, adducts) were optimized in a vacuum using the default thresholds of the program. The character of the stationary points was determined with analytical calculations of the second-derivative and all energy minima possessed only positive frequencies, while the TS were confirmed by the presence of one negative frequency. The latter was always visually inspected to check whether the displacement vector corresponds to the expected motion of the atoms in the TS connecting previously localized energy minimum structures. In the case of hydrogen atom abstraction mechanisms (H-abstraction), only the RC isomers with the lowest energy were considered (see SI for other possible RC and their ΔE values). The activation energy (dE^*‡*^) was always calculated as dE^*‡*^ = E (TS) – E (RC). All energies reported are zero-point energy (ZPE)-inclusive.

All XYZ coordinates of the vacuum optimized GLV structures are included in SI Section 6. Additionally, separate files for intermediates and products can be downloaded as a dataset zip folder available free of charge from the repository website RepOD at https://repod.icm.edu.pl/dataset.xhtml?persistentId=doi:10.18150/1J2I3C. The SI contains the Potential Energy Surface (PES) plots for the addition adducts, which were performed to confirm their lowest energy conformations.

Single-point calculations with implicit solvation were performed for the vacuum-optimized structures. The presence of a water medium was simulated using the conductor-like polarizable continuum model (CPCM) and a dielectric constant of 80.4 (Barone and Cossi, 1998). In this case, the ZPE correction was taken from the vacuum calculations as the analytical frequencies calculated in the presence of CPCM model may be questionable (Ho et al., 2010). Within the adapted scheme, it may happen (*vide infra*) that energies of some TS are very close to (or even below) the energies of reactants. This is primarily a sign that the vacuum barrier is already rather small (since the CPCM correction here is small) and that the TS structure has a pronounced ionic character. In principle one can perform calculations with a micro-solvated model, but this would require time-consuming and computationally-intensive sampling of the molecules' conformation space, which is beyond the scope of the present work.

The bond dissociation energies (BDEs) were obtained using eq. 1 (He et al., 2019; Otto et al., 2019).

(1) $$∆E_{\mathrm{bde}}\left(R-H\right)=\left[E\left(R\right)+\mathrm{ZPE}\left(R\right)+E\left(H\right)\right]-\left[E\left(\mathrm{GLV}\right)+\mathrm{ZPE}\left(\mathrm{GLV}\right)\right]$$

where GLV, R, and H denote the GLV compounds, GLV alkyl radicals, and the hydrogen atom, respectively. E and *ZPE* stands for the electronic energies and the zero-point energies of the GLV, R, or H, respectively.

## 3 Results and discussion

### 3.1 Product studies and mechanisms of •OH radicals reactions with PENTOL, HEXOL, and HEXAL

The aqueous-phase photo-oxidation reactions of GLVs (PENTOL, HEXOL, and HEXAL) initiated by ^•^OH radicals in aqueous phase were performed to study their possible product distribution and contribution to the SOA formation. No direct photo-degradation of PENTOL, and HEXOL was observed, because their absorbance below 290 nm is negligible. HEXAL, in contrast, absorbs light in the range of 290–400 nm (ε = 51–0.6 M^−1^ cm^−1^, c.f. SI Section 1) and was therefore isomerized to the *Z*-isomer within irradiation experiments.

The steady state concentration of •OH can be reported as 1 × 10^−13^ M for the oxidation reactions determined using the COmplex PAthway SImulator (COPASI) (Hoops et al., 2006) based on the approach developed by Otto et al. (2017) (see SI Section 3), which was within a 10^−12^ to 10^−15^ M range observed in the ambient (Herrmann et al., 2010). Moreover, the reaction of GLV with •OH radical competes with further HO_*x*_ side reactions (2, 3, and 4, Table S2) within the system. However, the turnover efficiency calculations using the COPASI model (see SI) show that the sink of •OH to the HO_x_ side reactions is always <20 %. Hence, their influence to the product yield is negligible.

The slopes of the linear ln [GLV]_t_/[GLV]_0_ *vs* time (h) plots gave the pseudo first-order rate constants for decay, whose standard errors were determined within 95 % confidence interval by regression analysis (SI Section 4). The calculated decay rates were (0.023 ± 0.005) h^−1^, (0.039 ± 0.004) h^−1^, and (0.029 ± 0.005) h^−1^, corresponding to the (13.6 ± 2.9)%, (23.3 ± 2.3)%, and (17.4 ± 3.1)% loss in 6 h for PENTOL, HEXOL, and HEXAL, observed within the molar fraction profiles (Figs. S3a, S6a, and S9b, respectively).

**Scheme 1:**
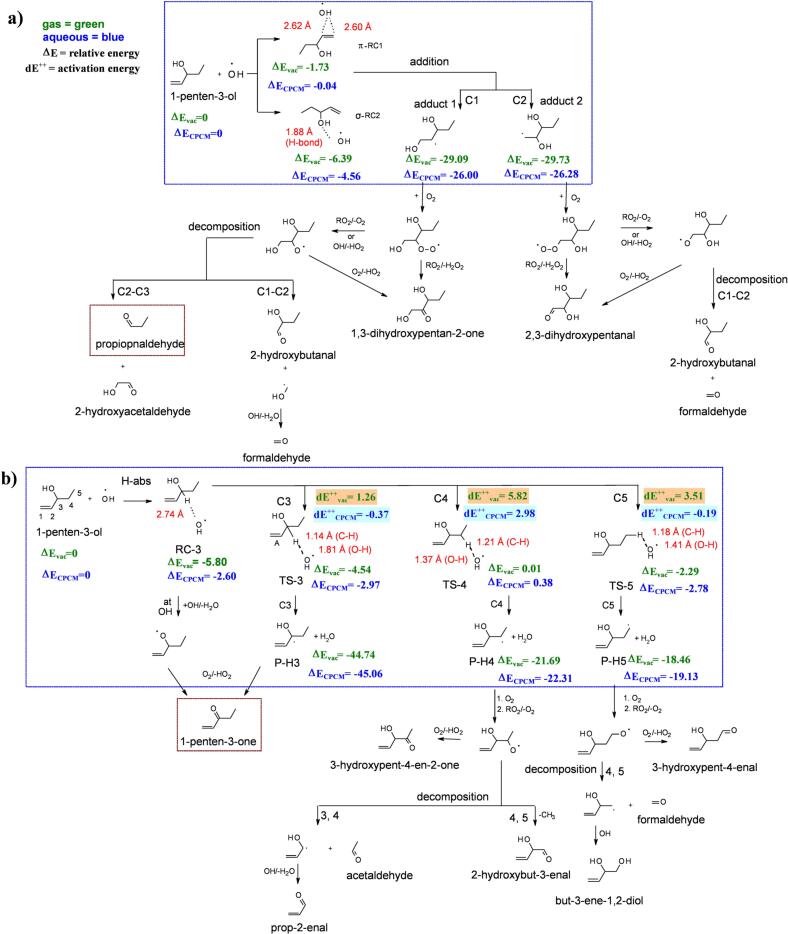
Proposed chemical mechanism and key products (in red boxes) for the reaction of 1-penten-3-ol (PENTOL) and •OH: a) addition channel, b) H-abstraction channel. 2-D scheme of the DFT optimized geometries with obtained energy (∆E and dE^*‡*^, in kcal mol^−1^) for each substrate is shown within blue dotted box, respectively.

**Scheme 2:**
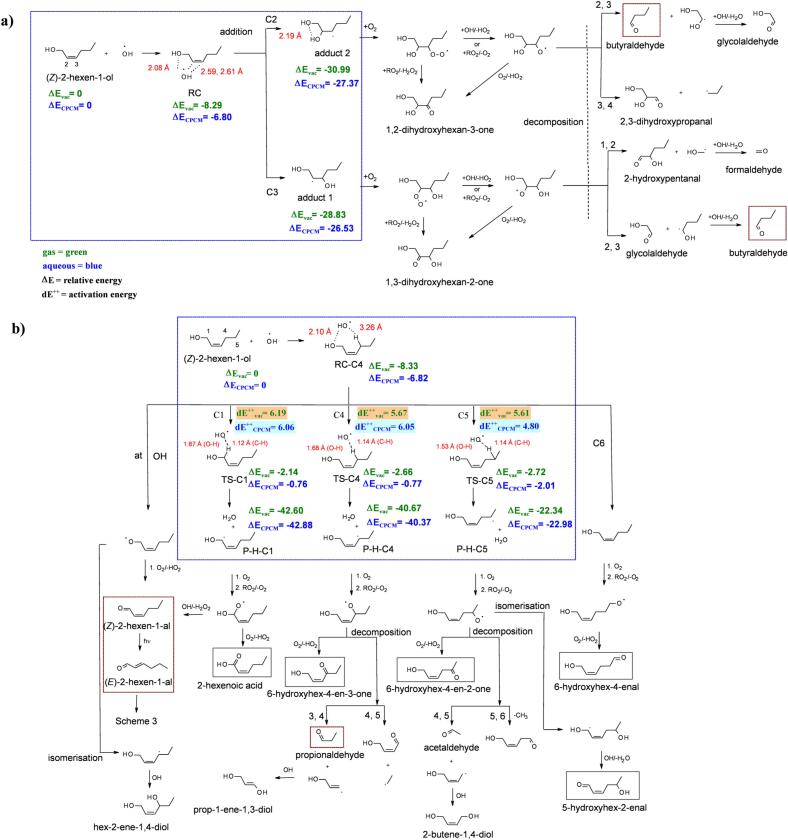
Proposed chemical mechanism and key products (in red boxes, and grey boxes for possible products detected with LC-HRMS as C_6_H_10_O_2_) for the reaction of *Z*-2-hexen-1-ol (HEXOL) and •OH: a) addition channel, b) H-abstraction channel. 2-D scheme of the DFT optimized geometries with obtained energy (∆E and dE^*‡*^, in kcal mol^−1^) for each substrate is shown within blue dotted box, respectively.

**Scheme 3:**
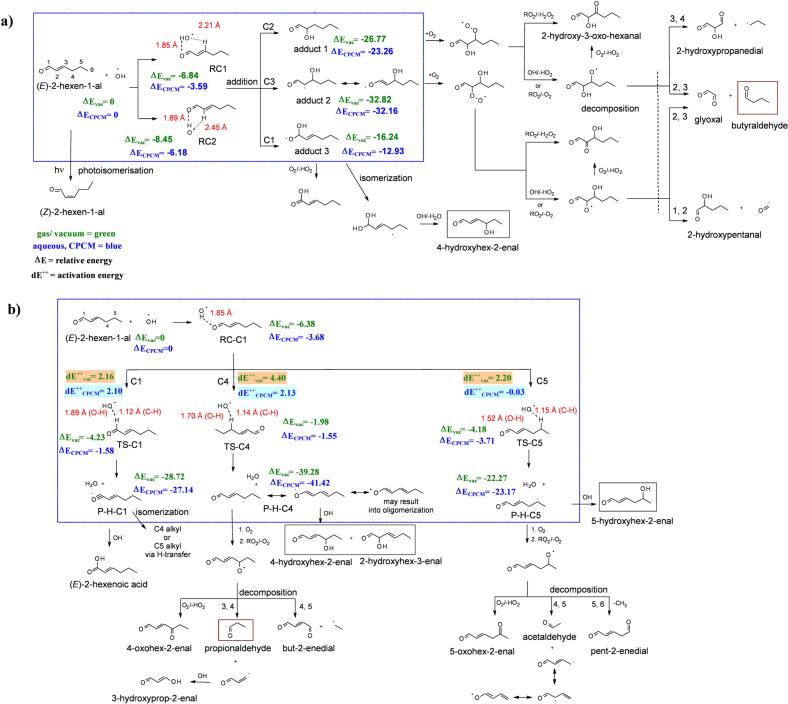
Proposed chemical mechanism and key products (in red boxes, and grey boxes for possible products detected with LC-HRMS as C_6_H_10_O_2_) for the reaction of (*E*)-2-hexen-1-al (HEXAL) and •OH: a) addition channel, b) H-abstraction channel. 2-D scheme of the DFT optimized geometries with obtained energy (∆E and dE^*‡*^, in kcal mol^−1^) for each substrate is shown within blue dotted box, respectively.

DFT results for all three selected GLV compounds are discussed in respective GLV sections below and shown using 2-D schemes (Scheme 1, Scheme 2, Scheme 3), where ∆E (kcal mol^−1^) is relative energy for each intermediate, *i.e.*, reaction complex (RC), addition adducts (adducts), transition states (TS), and hydrogen abstraction products (P—H, or P-H-C). We neither expected nor found any electronic barriers in the addition reactions of •OH to PENTOL, HEXOL, and HEXAL, because such reactions are analogous to bond formation between two atoms in vacuum. These findings are consistent with small experimental activation energies, *E*_*A*_ from 1.7 to 3.6 kcal mol^−1^ (Sarang et al., 2021a). The TS seems to play role only in the H-abs reactions. Overall DFT-calculated activation energies in the gas phase are small but positive (Scheme 1, Scheme 2, Scheme 3). The partial inclusion of solvent effects *via* CPCM scheme lowers all the barriers significantly. Moreover, in most cases one GLV alkyl/alkoxy radical product (adducts, P—H, or P-H-C) is energetically well separated from others and characterized with the lowest ∆E value. For PENTOL, the lowest dE^*‡*^ is found for TS-3, and the lowest ∆E for a P—H3 (Scheme 1b). Thus, the DFT determined kinetic (dE^*‡*^) and thermodynamic (∆E) values agree and indicate that the PENTOL reaction is rather thermodynamically controlled. However, for HEXOL (Scheme 2b) and HEXAL (Scheme 3b), dE^*‡*^ and ∆E values do not correlate. For HEXOL, the lowest dE^*‡*^ is for TS-C5 while the lowest ∆E – for P-H-C1 (Scheme 2b). For HEXAL, the lowest dE^*‡*^ is for TS-C5 and the lowest ∆E for P-H-C1 (Scheme 3b). In those cases, the kinetic and thermodynamic indicators do not agree and indicate that the reaction outcome might depend on temperature. The BDE calculation results provided in Table 1 are compared and discussed later with the DFT results for the H atom abstraction in each GLV.

**Table 1:** BDEs of C—H and O—H bond in PENTOL, HEXOL, and HEXAL calculated in vacuum and aqueous phase compared to experimentally obtained typical bond enthalpies (Blanksby and Ellison, 2003)*, kcal mol^−1^ at 298 K.

	H abstraction position	O-H	C1- H	C3-H	C4-H	C5-H	C6-H	Reference
GLV	Experiments	105 ^a^	^b^88	^c^97	99^e^	99 ^e^	101^f^	(Blanksby and Ellison, 2003)
				^d^89				
PENTOL	Vacuum	98		73	94	96		This study
	Aqueous	96		69	91	94		
HEXOL	Vacuum	97	74		78	94	97	This study
	Aqueous	97	73		78	93	97	
HEXAL	Vacuum		87		77	94	98	This study
	Aqueous		88		76	94	98	

*Experimental values for: ^a^ CH_3_O-H; ^b^ aldehydic group (H-CHO); ^c^ tertiary (3°) hydrogen H-C(CH_3_)_3_; ^d^ allylic hydrogen CH_2_CHCH_2_-H; ^e^ isopropylic hydrogen H-CH(CH_3_)_2_; ^f^ H-CH_2_CH_3_.

#### 3.1.1 Reactivity of PENTOL with •OH radicals

For the aqueous-phase reaction of PENTOL with •OH, two carbonyl products, *i.e.*, propionaldehyde and 1-penten-3-one, were clearly identified by cGC-MS (Scheme 1). The addition of •OH to PENTOL can occur either at C1 or C2 position (Scheme 1a) forming the alkyl radicals, followed by the addition of O_2_ forming the peroxy radicals. The latter can further combine with other peroxy radicals and form either H_2_O_2_ and the corresponding carbonyl compounds or O_2_ and the respective alkoxy radicals (von Sonntag and Schuchmann, 1991). Finally, the alkoxy radicals can undergo the intramolecular H-atom shift with the nearest -CH group forming a hydroxyl-alkyl radical, which can react with O_2_ to form an α-hydroxyl-peroxyl radical, and subsequently decomposes to the hydroperoxyl radical (HO_2_) and the corresponding carbonyl compound (Atkinson and Arey, 2003). Another reaction pathway of alkoxy radicals can be the decomposition to two corresponding carbonyl compounds (Scheme 1a). In the case of the H abstraction pathway (Scheme 1b), the •OH abstracts the H atom from C3, C4, C5 or -OH functional group in PENTOL to form the corresponding radicals, which further react with O_2_ following a mechanism similar to the addition channel.

**Fig. 1:**
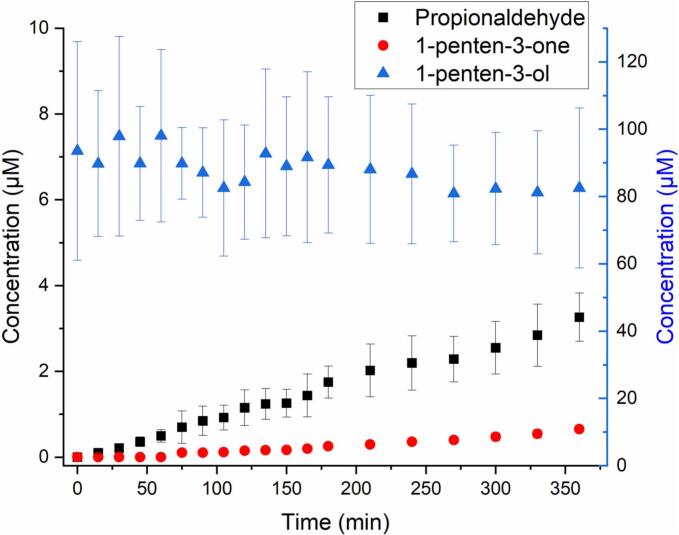
Photo-oxidation of 1-penten-3-ol initiated by OH radicals: concentration time profiles of 1-penten-3-ol (blue, right y-axis), propionaldehyde product (black, left axis), and 1-penten-3-one product (red, left y-axis). (For interpretation of the references to colour in this figure legend, the reader is referred to the web version of this article.)

The cGC-MS total ion chromatograms (TIC) for the reaction samples taken at 0 and 6 h are shown in SI Fig. S2. After 6 h, the molar fraction of PENTOL decreased by (12 ± 6)%, and an average (3.5 ± 0.6)% molar fraction of propionaldehyde, and (0.7 ± 0.2)% fraction of 1-penten-3-one formed (Fig. 1 and S3a). Fig. 1 shows the concentration-time profile of PENTOL during the photo-oxidation reaction, and the formation of propionaldehyde and 1-penten-3-one.

The cGC-MS method for alcohols could be improved with the addition of internal standards in future analyses and thus error bar could be lowered (PENTOL, here). To compensate, calibration curves with *R*^*2*^ > 0.99 were obtained at the end of each individual sample run for PENTOL.

DFT calculations (CPCM model, Scheme 1a) showed that due to the presence of H-bond between •OH and O atom of the -OH group of PENTOL, the σ-RC2 reaction complex was more stable in the aqueous phase by at least 4 kcal mol^−1^ than the π-RC1 reaction complex. Formation of both adducts 1 and 2 seems equally favorable in the aqueous phase (Scheme 1a). DFT calculated ∆E value shows adduct 2 with a slightly higher stability than adduct 1 (by 0.3 kcal mol^−1^). However, the intramolecular long-range interactions between two –OH groups are observed to be slightly stronger for adduct 1 (2.08 Å), than for adduct 2 (2.25 Å) and terminal radical is less preferable in general. Thus, the propionaldehyde formation in the experiments can be explained *via* addition at C1 (Scheme 1a). Other products of the addition channel might be below the detection limit for a total reaction time of 6 h and were therefore not detected by cGC-MS or LC-HRMS.

In Scheme 1b, the lowest dE^*‡*^_*vac*_ barrier is 1.26 kcal mol^−1^ for TS-3, which approaches zero within our simple CPCM correction model, essentially reflecting the initial (uncorrected) small value and pronounced charge asymmetry in the TS structure (stabilizing interaction with a polar solvent). The computed ∆E value for P—H3 in the solvent (Scheme 1b) shows that the H abstraction at C3 favors the formation of a stable allylic radical product (P—H3), which is more stable by at least 22–25 kcal mol^−1^ than other products P—H4 (C4), and P—H5 (C5). The BDE for hydrogen at C3 was the lowest (C3 < C4 < C5, see Table 1), and agrees with the ∆E values (Scheme 1b).

Overall, the alkyl radical product of H abstraction at the C3 site appears the most thermodynamically stable (∆E_CPCM_ = − 45.06 kcal mol^−1^, Scheme 1b), followed by the addition reaction at C1 and C2 (∆E_CPCM_ = −26.00, and − 26.28 kcal mol^−1^, Scheme 1a). However, the experimentally observed molar fraction of the addition product propionaldehyde is higher than that of 1-penten-3-one from the H abstraction (3:1), which is due to the barrierless pathway in the addition channel. Such a contradiction was also observed in previous DFT studies for the reaction of PENTOL with Cl (Rodriguez et al., 2010) where the product of the H abstraction at C3 was found to be thermodynamically more stable by at least 1.4 kcal mol^−1^ than other products.

For comparison, the major products detected in the gas-phase reaction of PENTOL with •OH included formaldehyde (35 ± 4)%, and glycolaldehyde (47 ± 6)% (Orlando et al., 2001). The reported average molar yield of the products from gas-phase ozonolysis of PENTOL: formaldehyde (49 ± 2)%, 2-hydroxybutanal (46 ± 3)% and propionaldehyde (15 ± 2)% (O'Dwyer et al., 2010), while formaldehyde, propionaldehyde and 2-hydroxybutanal were also found in other studies (Grosjean and Grosjean, 1995; Grosjean and Grosjean, 1997). Furthermore, propionaldehyde and 1-penten-3-one are the reported products of reaction between PENTOL and Cl radicals (Rodriguez et al., 2010). The other possible products suggested in Scheme 1 and reported within the gas-phase studies, such as formaldehyde, 2-hydroxybutanal and acetaldehyde, could not be detected in the aqueous-phase samples, possibly due to low concentrations produced in the 6 h reaction. In total, those undetected products might fill the 2.4–13 % gap between the loss of PENTOL and formation of propionaldehyde and 1-penten-3-one. We had not observed any loss of PENTOL during six-hour photolysis in the absence of H_2_O_2_ and dark oxidation with H_2_O_2_ (no UV light) (Fig. S3b). Photolysis of PENTOL in actinic region is insignificant, and the main sink for PENTOL is the reaction with •OH, while the O_3_ and NO_3_• reactions are also insignificant (Jimenez et al., 2009a).

#### 3.1.2 Reactivity of HEXOL with •OH radicals

**Fig. 2:**
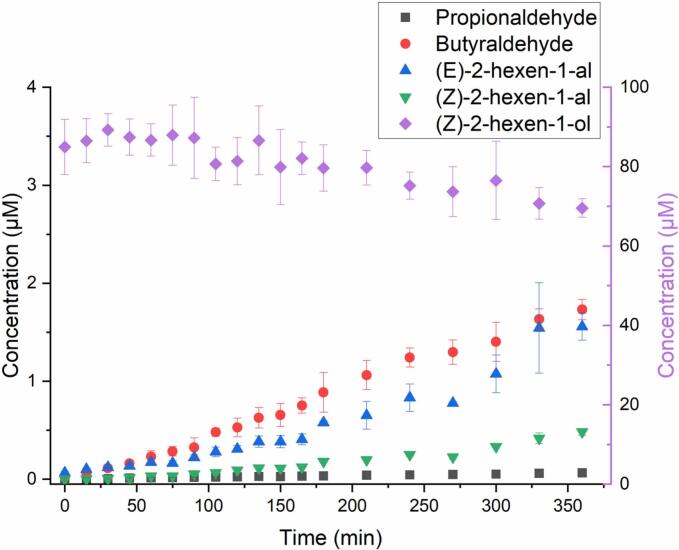
Photo-oxidation of (*Z*)-2-hexen-1-ol initated by OH radicals: concentration time profiles of (*Z*)-2-hexen-1-ol (right y-axis), propionaldehyde, butyraldehyde and (*E*)-2-hexen-1-al, and (*Z*)-2-hexen-1-al formed (all products, left y-axis).

The reaction of HEXOL with •OH was investigated using both cGC-MS and LC-HRMS. Fig. S4 shows the cGC-MS TIC of the reaction samples (at 0 h and 6 h, respectively) with peaks corresponding to the identified reactants, while Fig. S5 shows the TIC obtained for HEXOL using UHPLC in PDA/UV mode. After 6 h, up to (20 ± 3)% molar of HEXOL was converted to the oxidation products (Fig. 2 and S6a). Using the cGC-MS analysis, the following carbonyls (in molar fraction): propionaldehyde (0.08 ± 0.01)%, butyraldehyde (2.0 ± 0.1)%, HEXAL (1.8 ± 0.2)%, and (*Z*)-2-hexen-1-al (*Z*-HEXAL, 0.57 ± 0.04)% were confirmed in the reaction samples after 6 h (Fig. S6a). The rationale for the proposed reaction mechanism for the HEXOL-OH system (Scheme 2) is similar to that of the PENTOL-OH system explained in the previous section.

The LC-HRMS analyses in negative ion mode revealed the *m/z* 113.06080 [M-H]^−^ ions with formula C_6_H_9_O_2_. The extracted ion chromatogram (EIC, Fig. S7) revealed the compound was absent at the initial stage of the experiment (*t* = 0 h), and appeared at *t* = 6 h (RT = 6.6 min). The signal intensity profile showed a continuous increase in the concentration of the novel C_6_H_10_O_2_ product as *m/z* 113.06080 [M-H]^−^ ions (Fig. S8). Based on the proposed mechanism (Scheme 2b, Scheme 3), it can represent hydroxy hexenal, hexenoic acid or hydroxy hexenone, which form either *via* the HEXAL pathway (Section 3.1.3) or *via* the H abstraction at C4 and C5 sites in HEXOL. The analysis of authentic standards showed *m/z* 113.06080 [M-H]^−^ was not 2-hexenoic acid. Hence, the C_6_H_10_O_2_ product is either hydroxy hexenal or hydroxy hexenone possessing more acidic hydrogen than hexenoic acid. However, since no authentic standards were available for this work, further efforts are required to confirm either of the structures.

The formation of butyraldehyde possibly occurs *via* the •OH addition at C=C double bond of HEXOL (either C2 or C3 position), followed by the decomposition of the corresponding HEXOL-based alkoxy radicals (Scheme 2a). Whereas, the H abstraction channel leads to the formation of two other products, propionaldehyde and (*E*/*Z*)-2-hexen-1-al (Scheme 2b).

The addition is more stable at C2 than at C3 by at least 1 kcal mol^−1^ in the aqueous phase (Scheme 2a). Adduct 2 is free of any stearic hindrance. However, the resulting long-range intramolecular interactions between O and H atoms of two adjacent -OH groups ((d_O…H_ = 2.19 Å, Scheme 2a) in adduct 1 are an additional stabilizing factor. Thus, adducts 1 and 2 can equally lead to the formation of experimentally observed butyraldehyde.

The product of the H abstraction at the C1 site (P-H-C1) is the most stable (∆E_CPCM_, −42.88 kcal mol^−1^, Scheme 2b), followed by the P-H-C4 (∆E_CPCM_, −40.37 kcal mol^−1^, Scheme 2b). This finding can be explained by the formation of a stable allylic radical product (P-H-C1, and P-H-C4, Scheme 2b) and confirms the formation of propionaldehyde and (*E/Z*)-2-hexenal (Scheme 2b) observed in the photo-oxidation experiments. These results agree with the BDE calculations (C1 < C4 < C5, Table 1). However, the reaction activation barrier (dE^*‡*^_CPCM_ = 4.80 kcal mol^−1^) for H abstraction was the lowest at C5 (RC-C4 to TS-C5, Scheme 2b), which supports theoretically the formation of the experimentally observed C_6_H_10_O_2_ *via* the hydrogen-transfer or isomerization of the HEXOL-alkoxy radical, as proposed in Scheme 2.

Overall, the DFT results showed that the products of H abstraction at C4 and C1 are more stable and favorable by at least 15 kcal mol^−1^ compared to the addition products. Gai et al. (2015) also emphasized the importance of the H abstraction channels in the gas-phase reactions of hexenols. Nevertheless, the addition reactions are barrierless (DFT) and fast enough (GCM, SI, *k* = 9.1 × 10^9^ L mol^−1^ s^−1^) to explain butyraldehyde as the major product observed, while the H abstraction channel is slower, but non-negligible and results in the formation of propionaldehyde, (*E*/*Z*)-2-hexen-1-al, and C_6_H_10_O_2_.

For comparison, Gibilisco et al. (2013) reported butyraldehyde as the major oxidation product of the gas-phase reaction between 2-hexen-1-ol and •OH. In the case of 3-hexen-1-ol, propionaldehyde was reported as the major oxidation product. The reaction of (*E*)-2-hexen-1-ol occurred mainly by the addition of •OH to the double bond, resulting in the formation of corresponding aldehyde and alcohol following the C2-C3 decomposition pathway (Gibilisco et al., 2013).

#### 3.1.3 Reactivity of HEXAL with OH radicals

**Fig. 3:**
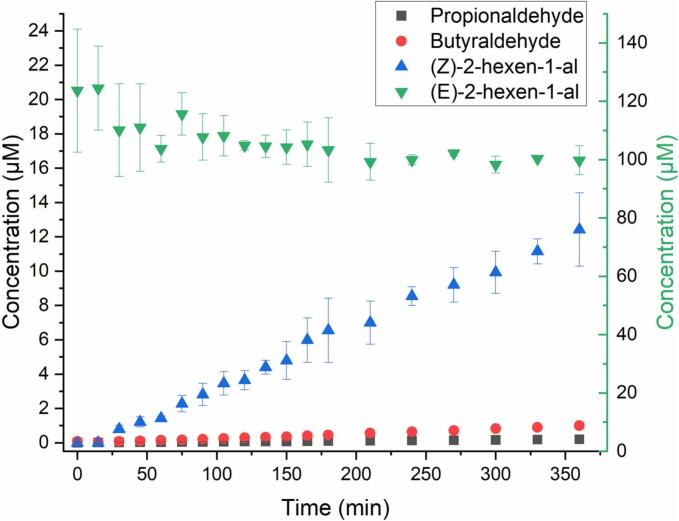
Photo-oxidation of (*E*)-HEXAL initiated by OH radicals: concentration time profiles of (*E*)-HEXAL (right y-axis) and key products, propionaldehyde, butyraldehyde, and (*Z*)-HEXAL (left y-axis).

In contrast to PENTOL and HEXOL, HEXAL shows a considerable light absorption in the near UV (290–400 nm wavelengths, see SI Section 1), and its photo-isomerization produced 10–12 % molar of *Z*-HEXAL (Figs. 3 and S11b). HEXAL can also be oxidized in the aqueous phase by addition or H abstraction (Scheme 3). Alkyl radicals formed by the H abstraction at the C4 site can also oligomerize at later stages. However, products of that aging could not be identified, most likely due to extremely low concentrations even after 6 h.

During the aqueous-phase photo-oxidation, (18.5 ± 9.9)% molar of HEXAL decomposed to butyraldehyde (0.81 ± 0.02)% and propionaldehyde (0.162 ± 0.004)%, and photo-isomerized to *Z*-HEXAL (10 ± 0.01)% (Figs. 3 and S9b). The concentration and molar percent profiles for HEXAL (Figs. 3 and S9b) were calculated using cGC-MS data, while it could also be detected using the HPLC-PDA/UV (Fig. S10).

Similar to HEXOL, the C_6_H_9_O_2_ ion (*m/z* 113.06080 [M-H]^−^) was detected in the reaction samples of HEXAL reaction solutions using the LC-HRMS analysis (Figs. S12 and S13). Analyzing the results for those two GLVs and the proposed reaction mechanisms (Scheme 2, Scheme 3), the C_6_H_10_O_2_ formula was assigned to hydroxy hexenal rather than hydroxy hexenone. Furthermore, the relative abundance of *m/z* 113.06080 [M-H]^−^ ion was by two orders of magnitude higher in the HEXAL reaction sample than HEXOL, and occurred earlier. The signal intensity-time profile for *m/z* 113.06080 [M-H]^−^ is shown in Fig. S13b. Thus, most of the unexplained loss of for HEXAL (up to 8 % molar) could be due to the formation of C_6_H_10_O_2_.

The product of OH addition at C3 (adduct 2) is more stable than at C2 (adduct 1) by at least 9 kcal mol^−1^, and results in the formation of butyraldehyde (Scheme 3a). Both proposed reaction complexes (RC1 and RC2) are stabilized by relatively strong intermolecular hydrogen bonds (Scheme 3a). The addition of •OH at the carbonyl group (adduct 3) is the least favorable and unlikely to occur.

Due to the formation of a stable allylic radical in P-H-C4 (Scheme 3b) with a low energy barrier (dE^*‡*^_CPCM_ = 2.13 kcal mol^−1^) the H abstraction at C4 produces the most stable radical product (∆E_CPCM_, −41.42 kcal mol^−1^). This is followed by the H abstraction at aldehydic C1 site, based on the ∆E_CPCM_ (−27.14 kcal mol^−1^) of P-H-C1. These results agree well with the order of BDE values (C4 < C1 < C5, Table 1). The activation energy barrier for TS-C1, dE^*‡*^_CPCM_ = 2.10 kcal mol^−1^ is slightly lower than that for TS-C4. This suggests that the isomerization (Scheme 3b) due to hydrogen transfer to C4 and C5 sites increases the stability of P-H-C1 compared to P-H-C5 (the least stable product in terms of ∆E).

Overall, the H abstraction at C4 in HEXAL gives the most stable product by at least 9 kcal mol^−1^ compared to the addition products, and leads to the formation of propionaldehyde as well as C_6_H_10_O_2_ product(s). Moreover, the lowest dE^*‡*^_vac_ for TS-C1 can also explain the formation of C_6_H_10_O_2_ *via* the formation of P-H-C1 (Scheme 3). Two distinct H abstraction pathways (C4 and C1) leading to the formation of C_6_H_10_O_2_ may account for >5 % missing molar fraction loss of the HEXAL reported, and make C_6_H_10_O_2_ the most important product. A more stable addition product at a C3 site proves the formation of butyraldehyde during the photo-oxidation experiments.

Gas-phase reaction of HEXAL with OH has not been studied, while the reaction with O_3_ produced glyoxal (0.56 ± 0.04) and butyraldehyde (0.53 ± 0.06) yield fractions (Grosjean et al., 1996b).

### 3.2 Atmospheric significance and impact of aqueous-phase GLVs transformation pathway and their oxidation products

Short chain carbonyl compounds, such as propionaldehyde and butyraldehyde identified in this work, play a key role in the atmospheric photochemical processes (Grosjean et al., 1993; Baez et al., 2003). They belong to the first oxidation products of the primary emitted hydrocarbons, which react with atmospheric radicals, such as •OH (Granby et al., 1997). Additionally, they can photolyze and produce •OH radicals in the atmosphere (Committee on Aldehydes et al., 1981). In the presence of NO_*x*_, they can lead to the formation of peroxy acetyl nitrate, nitric acids, carboxylic acids, and other aerosol-bound components *via* photolysis, gas and aqueous phase reactions (Veyret et al., 1989), eventually leading to the formation of photochemical smog (Carlier et al., 1986; Grosjean, 1988; Bakeas et al., 2003). The ambient air concentrations of propionaldehyde (0.6–3.0 ppb) and butyraldehyde (0.7–2.4 ppb) are already reported (Grosjean et al., 1996a; Possanzini et al., 1996).

The potential of GLV oxidation and their products to contribute to aqueous SOA (aqSOA) formation depends on the GLV partitioning into the aqueous phase and the products' tendency to remain therein. The EPI suite 2012 from EPA (US_EPA, 2012) was used to obtain the estimated and experimentally available literature data (if available) based on physical properties of the GLV compounds, and their oxidation products (Table S5). The data provide insight into the tendency of the oxidation products to remain in the aqueous phase and contribute to aqSOA formation. Properties estimated by the EPI suite, such as the Henry's law constant (HLC) and vapor pressure (VP), varied by several orders of magnitude depending on the method used for the estimation (Meylan and Howard, 1991; Boethling et al., 2004; Pankow and Asher, 2008; Hansel et al., 2015). The bond estimation method provided good values of HLC and VP, which agreed well with the experimental values for propionaldehyde, and butyraldehyde (Table S5). The available experimental data for GLVs are very limited, and require future attention to better assess the relevance of such compounds for atmospheric reactions in the aqueous phase (Sarang et al., 2021b). Estimated HLC for all oxidation products are lower than those for the parent compounds, except the C_6_H_10_O_2_ product whose HLC is higher by at least three order of magnitude. For simplicity, we estimated the values only for one of the proposed isomers, *i.e.*, 4-hydroxy-2-hexenal (Table S5), as the results for other isomers were practically produced the same. Partitioning of volatiles between the aqueous and gas phases in atmospheric systems depends on Henry's constant and the liquid water contents of the system (LWC). The significant aqueous-phase concentrations of PENTOL, HEHOL, and HEXAL occur at LWC higher than for the atmospheric aqueous systems (Fig. S21). However, chemical reactions accompanying the uptake can significantly increase the amounts of GLV transferred into the aqueous phase. The reasonable partitioning of GLV oxidation products between phases requires similar LWC (Fig. S21) in concordance with the estimated VPs being even higher than those of the parent GLVs. Only the C_6_H_10_O_2_ product has higher aqueous-phase partitioning tendency, which is at least three orders of magnitude higher than that of the parent GLV and other oxidation products. It may reside more in aqueous phase, and hence effectively contribute to the aqSOA. However, the potential of the less soluble oxidation products to form aqSOA might increase by further chemical reactions with oxidants in the aqueous phase. That problem definitely requires further research. Because of the higher O:C ratio in C_6_H_10_O_2_ than in the parent GLVs (Schemes S2 and S3), C_6_H_10_O_2_ could potentially contribute to aqSOA. The unsaturated carbonyl products identified in this study (1-penten-3-one, hydroxy hexenal, hydroxy hexenone) can potentially contribute to the formation of novel organosulfates and organonitrates in the atmosphere (Schone et al., 2014; Sareen et al., 2016; Wach et al., 2019; Bruggemann et al., 2020).

## 4 Conclusions

In the present study, we investigated the potential of selected GLVs, *i.e.*, PENTOL, HEXOL, and HEXAL to form SOA *via* the aqueous-phase oxidation by •OH radicals. Propionaldehyde, butyraldehyde, 1-penten-3-one, 2-hexen-1-al, and a new compound of molecular formula C_6_H_12_O_2_ were identified as the oxidation products of the reactions. Propionaldehyde and butyraldehyde may further react with •OH, O_3_ and other oxidants affecting air quality and human health under conditions with higher GLV concentrations. The new carbonyl product with molecular formula C_6_H_10_O_2_ formed during the reactions of HEXOL and HEXAL with •OH can potentially contribute to the formation of aqSOA, so its complete identification will be a part of future work while other carbonyls are likely the starting point towards the formation of aged SOA originating from GLVs PENTOL, HEXOL, and HEXAL.

Complementary to the experimental findings, a theoretical study using DFT was carried out to investigate and understand the importance of addition and H abstraction mechanistic pathways of the reactions between the selected GLVs and •OH radicals. It is known, that the addition reaction pathway is barrierless and dominant, however DFT findings showed that some H abstraction pathways should not be neglected, especially those leading to a new C_6_H_10_O_2_ compound.

For future work, the study of their reactions at air-water interface is highly recommended to obtain complete picture of their contribution to aqSOA. In addition, it would be important to determine experimentally the GLVs partition coefficients between air and the aqueous phases to resolve the uncertainties obtained with estimation.

## CRediT authorship contribution statement

**Kumar Sarang:** Conceptualization, Investigation, Formal analysis, Data curation, Writing – original draft. **Tobias Otto:** Methodology, Investigation. **Sahir Gagan:** Conceptualization, Methodology, Investigation, Formal analysis. **Krzysztof Rudzinski:** Conceptualization, Methodology, Investigation, Formal analysis, Writing – review & editing. **Thomas Schaefer:** Supervision, Formal analysis, Writing – review & editing. **Martin Brüggemann:** Investigation. **Irena Grgić:** Conceptualization, Writing – review & editing. **Adam Kubas:** Supervision, Methodology, Investigation, Formal analysis, Writing – review & editing. **Hartmut Herrmann:** Conceptualization, Supervision, Writing – review & editing. **Rafal Szmigielski:** Supervision, Conceptualization, Investigation, Writing – review & editing.

## Declaration of competing interest

The authors declare no competing financial interest. All data to support the conclusions of this manuscript are included in the main text and Supporting Information.

## Data Availability

Data will be made available on request.

## Appendix A Supplementary data

Supplementary Information Image 1
