# Supplementary material for: Aqueous-phase photo-oxidation of selected green leaf volatiles initiated by •OH radicals: Products and atmospheric implications
Source: Sci Total Environ. 2023 Jun 25;879:162622. doi: 10.1016/j.scitotenv.2023.162622 (PMC10199410; doi:10.1016/j.scitotenv.2023.162622)
Supplement: Supplementary file 1 — Supplementary Information [file mmc1.docx]

**Supplementary Information**

**Aqueous-phase Photo-oxidation of Selected Green Leaf Volatiles initiated by** ^•^**OH radicals: Products and Atmospheric Implications**

Kumar Sarang ^a^, Tobias Otto ^b, d^, Sahir Gagan ^a, e^, Krzysztof Rudzinski ^a^, Thomas Schaefer ^b^, Martin Brüggemann ^b, f^, Irena Grgić ^c^, Adam Kubas ^a,^ *, Hartmut Herrmann ^b,^ *, Rafal Szmigielski ^a,^ *

*^a^ Institute of Physical Chemistry Polish Academy of Sciences, 01-224 Warsaw, Poland;*

*^b^ Atmospheric Chemistry Department, Leibniz Institute for Tropospheric Research, 04318, Leipzig, Germany*

*^c^ Department of Analytical Chemistry, National Institute of Chemistry, SI-1000, Ljubljana, Slovenia;*

*^d^ Presently: Labor für Wasser und Umwelt GmbH, Berliner Str. 13, 04924, Bad Liebenwerda;*

*^e^ Presently: Department of Atmospheric Sciences, Texas A&M University, College Station, TX 77843, United States;*

*^f^ Presently: Bayer AG, Crop Science Division, R&D, Environmental Safety, Alfred-Nobel-Straße 50, 40789 Monheim am Rhein, Germany*

________________________

* Corresponding authors. *E-mail addresses:* ralf@ichf.edu.pl (R. Szmigielski), akubas@ichf.edu.pl (A. Kubas), herrmann@tropos.de (H. Herrmann)

Table of Contents

1. UV SPECTROSCOPY DATA 3

2. EXPERIMENTAL SETUP 3

Fig. S1. The photo-oxidation reaction setup used for the present study. 4

Table S1. Details of the product study experiments at 298 K. 4

3. COPASI model 5

Table S2. COPASI estimation model used to determine steady state ^•^OH concentrations. 5

4. PSEUDO FIRST ORDER RATE OF DECAY 6

5. PHOTO-OXIDATION EXPERIMENTS 6

*cGC-MS and LC-HRMS analyses* 6

Table S3. Type of analysis, retention times, molar masses, and characteristic fragments. 8

*5.1. PHOTOOXIDATION OF PENTOL IN PRESENCE OF ^•^OH*  9

*5.2 PHOTOXIDATION OF HEXOL IN PRESENCE OF ^•^OH* 11

*5.3 PHOTOOXIDATION OF HEXAL IN PRESENCE OF ^•^OH.* 16

6. DENSITY FUNCTIONAL THEORY 21

Fig. S14. Vacuum optimized geometries of green leaf volatile compounds (GLVs) 21

Table S4. XYZ coordinates of vacuum optimized structures of selected GLVs 22

Fig. S15. Potential energy scans for the PENTOL adduct. 24

Fig. S16. Other possible higher energy RC for a H-abstraction pathway of PENTOL 25

Fig. S17. Potential energy scans for the HEXOL adduct 25

Fig. S18. Other possible higher energy RC for a H-abstraction pathway of HEXOL 25

Fig. S19. Potential energy scans for the HEXAL adduct 26

Fig. S20. Other possible higher energy RC for a H-abstraction pathway of HEXAL 26

7. ATMOSPHERIC IMPLICATIONS 27

Table S5. Physical properties of GLVs and observed oxidation products at 298 K 27

*Oxidation product partitioning between gas and aqueous phases* 28

Fig. S21. Gas-aqueous partitioning of GLV-OH oxidation products 28

8. REFERENCES 29

# **1. UV SPECTROSCOPY DATA**

UV spectra of the GLVs (1-penten-3-ol, (*Z*)-2-hexen-1-ol, and (*E*)-2-hexen-1-al) were presented in a recent review (Sarang et al., 2021). The molar absorption coefficients were used to predict the photolysis of the compounds in the actinic region. The dataset can be downloaded from the RepOD: ICM based repository website https://repod.icm.edu.pl/dataset.xhtml?persistentId=doi:10.18150/7LEJDC, and provides molar absorption coefficients (ε) for the range from 210 – 400 nm, with uncertainty ∆ε as the standard deviation obtained from ten measurements (five different concentrations, two measurements per each concentration).

# **2. EXPERIMENTAL SETUP**

A 300 mL custom made glass photo-reactor was used to perform GLVs photo-oxidation product studies in the presence of ^•^OH radicals (Fig. S1). The reactor was temperature controlled (Julabo F10 and Julabo HC) to maintain a constant temperature of 298 K and magnetically stirred (IKA^®^ RH basic 2). A quartz window sealed with two aluminum rings served as the optical window.

The LOT Quantum Design solar simulator (LSO805) with a 450 W ozone-free high-pressure xenon arc light lamp (LSB551) were used as UV light sources. To mimic the sunny daylight conditions, the light was filtered with AM1.5 filters, which allowed light with a wavelength above λ =290 nm only. Thus, any short wavelength photolysis of the species in the reactor was avoided. Otto et al. (2019) compared the lamp spectrum to the AM0 and AM1.5G sunlight spectra. The reactor setup for product studies is depicted in Fig. S1.


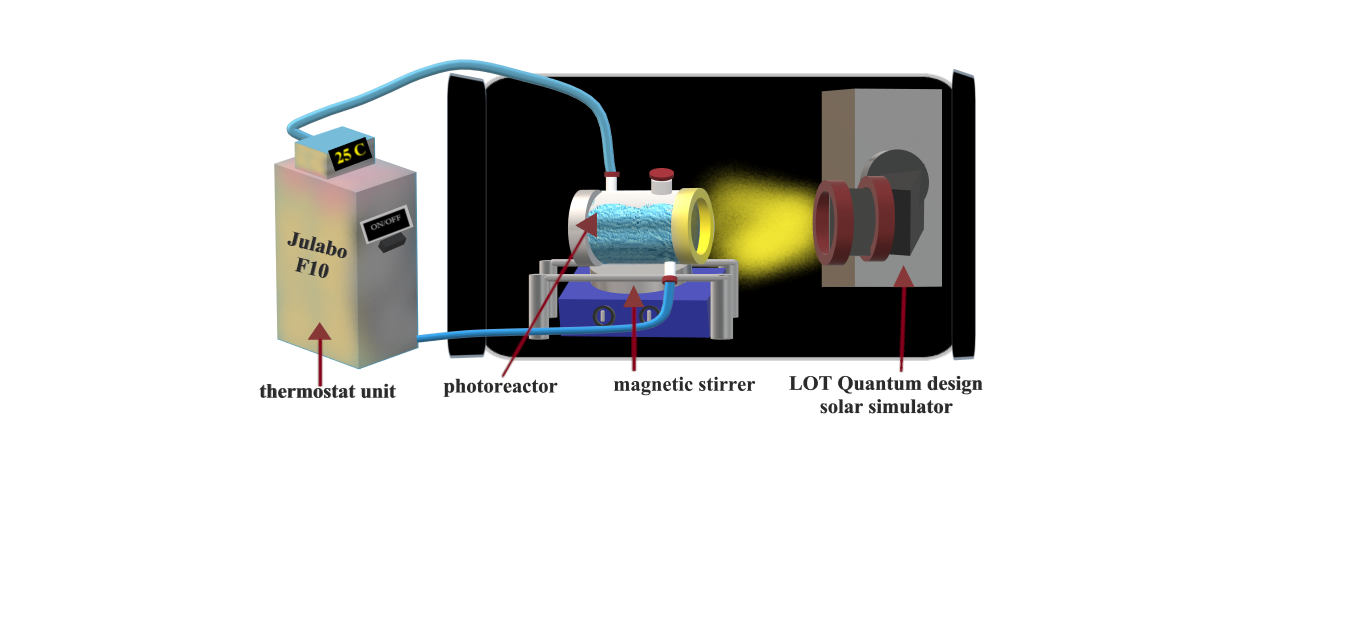


## **Fig. S1.** The photo-oxidation reaction setup at TROPOS, Leipzig used for the present study.

## **Table S1.** Details of the product study experiments at 298 K.

| **S. No.** | **GLV** | **Type of experiment** | **UV light** | **[GLV],**  **M** | **[H_2_O_2_],**  **M** | ***Repetition sets,**  ***n*** |
| --- | --- | --- | --- | --- | --- | --- |
| **1** | PENTOL | Dark H_2_O_2_ oxidation | No | $\text{1×}\text{10}^{\text{-4}}$ | $\text{5×}\text{10}^{\text{-3}}$ | 1 |
| **2** |  | Photolysis | Yes | $\text{1×}\text{10}^{\text{-4}}$ | - | 1 |
| **3** |  | ^•^OH oxidation | Yes | $\text{1×}\text{10}^{\text{-4}}$ | $\text{5×}\text{10}^{\text{-3}}$ | 2 |
| **4** | HEXOL | Dark H_2_O_2_ oxidation | No | $\text{1×}\text{10}^{\text{-4}}$ | $\text{5×}\text{10}^{\text{-3}}$ | 1 |
| **5** |  | Photolysis | Yes | $\text{1×}\text{10}^{\text{-4}}$ | - | 1 |
| **6** |  | ^•^OH oxidation | Yes | $\text{1×}\text{10}^{\text{-4}}$ | $\text{5×}\text{10}^{\text{-3}}$ | 2 |
| **7** | HEXAL | Dark H_2_O_2_ oxidation | No | $\text{1×}\text{10}^{\text{-4}}$ | $\text{5×}\text{10}^{\text{-3}}$ | 1 |
| **8** |  | Photolysis | Yes | $\text{1×}\text{10}^{\text{-4}}$ | - | 1 |
| **9** |  | ^•^OH oxidation | Yes | $\text{1×}\text{10}^{\text{-4}}$ | $\text{5×}\text{10}^{\text{-3}}$ | 2 |

*Total duration of each experiment was 6 h.

# **3. COPASI model**

The COmplex PAthway SImulator of biochemical systems (COPASI from Bioinformatics (Hoops et al., 2006), was used to calculate the initial OH concentration and its turnover efficiency with in the reaction system. The deterministic ordinary differential equation solver (LSODA) (Hindmarsh, 1983; Petzold, 1983; Hoops et al., 2006) was chosen for the reaction time courses simulation. Details of COPASI are also described elsewhere (Schaefer et al., 2012; Otto et al., 2017; Sarang et al., 2021).

## **Table S2.** Initial reactant concentrations and reactions considered in the COPASI estimation model used to determine steady state ^•^OH concentrations.

| No. | Reaction | *k* (298 K) | References | Species | [Conc.]_0_, M |
| --- | --- | --- | --- | --- | --- |
| 1 | H_2_O_2_ → OH + OH | 2.8 × 10^-6^ s^-1^ | (Otto et al., 2017) | H_2_O_2_ | 5.0 × 10^-3^ |
| 2 | OH + OH → H_2_O_2_ | 3.6 × 10^9^ L mol^-1^ s^-1^ | (Elliot and Buxton, 1992) | OH | 0 |
| 3 | OH + H_2_O_2_ → H_2_O + HO_2_ | 2.7 × 10^7^ L mol^−1^ s^−1^ | (Christensen et al., 1982) | H_2_O | 55.37 |
| 4 | OH + HO_2_ → H_2_O + O_2_ | 6.0 × 10^9^ L mol^−1^ s^−1^ | (Elliot and Buxton, 1992) | HO_2_ | 0 |
| 5 | OH + O_2_^−^ → OH^−^ + O^2^ | 1.1 × 10^10^ L mol^−1^ s^−1^ | (Christensen et al., 1982) | O_2_ | 2.6 × 10^-4^ |
| 6 | H_2_O ⇌ H^+^ + OH^−^ | → 0.0014 s^−1^,  ← 1.4 × 10^11^ L mol^−1^ s^−1^ | (Pastina and LaVerne, 2001) | O_2_^−^ | 0 |
| 7 | HO_2_ ⇌ O_2_^−^ + H^+^ | → 1.4 × 10^6^ s^−1^,  ← 5.0 × 10^10^ L mol^−1^ s^−1^ | (Pastina and LaVerne, 2001) | OH^−^ | 1.0 × 10^-7^ |
| 8 | GLV + OH → Products  **a.** 1-penten-3-ol;  **b.** (*Z*)-2-hexen-1-ol;  **c.** (*E*)-2-hexen-1-al | a. 6.3 × 10^9^ L mol^−1^ s^−1^ | (Sarang et al., 2021) | H^+^ | 1.0 × 10^-7^ |
|  |  | b. 6.7 × 10^9^ L mol^−1^ s^−1^ |  | GLV | 1.0 × 10^-4^ |
|  |  | c. 4.8 × 10^9^ L mol^−1^ s^−1^ |  | Products | 0 |

# **4. PSEUDO FIRST ORDER RATE OF DECAY**

The rate of overall GLV loss calculated based on the equation S1 (Richards-Henderson et al., 2014; Hansel et al., 2015), constituting loss due to reaction with OH ($k_{OH+GLV}\left[ OH \right]\left[ GLV \right]$), direct photolysis ($j_{GLV}\left[ GLV \right]$), and all other unknown loss pathways ($k_{other}\left[ GLV \right]$) :

$-\frac{d\left[ GLV \right]}{dt}=k_{OH+GLV}\left[ OH \right]\left[ GLV \right]+j_{GLV}\left[ GLV \right]+k_{other}\left[ GLV \right]$ (S1)

Given that, the [OH] is in the steady state, as estimated using COPASI model in this work, the equation one can be simplified to equation S3 upon combination of equation S1 & S2 followed by its integration.

$k_{GLV}^{'}=k_{OH+GLV}\left[ OH \right]+j_{GLV}+k_{other}$ (S2)

$ln\frac{\left[ GLV \right]_{t}}{{[GLV]}_{0}}={-k}_{GLV}^{'}\times t$ (S3)

# **5. PHOTO-OXIDATION EXPERIMENTS**

## *cGC-MS and LC-HRMS analyses*

**Carbonyl-targeted cGC-MS analyses.** An Agilent 7890 series GC system coupled with Agilent 5977B MSD, an Electron Ionisation Quadrupole Mass Spectrometer (EI-MS) and Agilent Technologies 7693 Auto sampler was used. Separation by GC was performed using HP-5MS UI Column (Agilent J&W GC columns 19091S-433, 30 m × 0.25 mm × 0.25 µm), in the splitless mode with an inlet temperature of 250°C. The GC separation was performed at following conditions: gas flow 1.2 mL min^-1^, inlet temperature program: 70°C isothermal for 0.41 min, first increased to 325°C with 600 K min^-1^, followed by achieving 350°C where it was held for 10 min. The column temperature was: 50°C for 2 min and elevated to 210°C with 10°C min^-1^. It was held at 210°C for 7 min, and was followed by post run at 320°C, held constant for 10 min.

**Alcohol-targeted cGC-MS analyses.** The samples were analyzed using an Agilent 6890 A series GC system coupled with Agilent 5973 N MSD, an Electron Ionization Quadrupole Mass Spectrometer, and Agilent Technologies 7683 Autosampler. A Phenomenex ZB-WAXplus column (30 m × 0.25 mm × 0.25 µm) with a gas flow of 1.2 mL min^-1^ was used for separation. The inlet was operated in the splitless mode with an inlet temperature program: 140°C isothermal, 40°C for 4 min, increased to 200°C with 10°C min^-1^, followed by a 5 min post-run at 230°C. The column temperature was 40°C isothermal for 4 min and then elevated to 200°C with 10°C min^-1^, where it was held constant for 5 min and ended.

**LC-HRMS analyses.** The LC-MS settings applied for the analysis were as follows: the UHPLC instrument equipped with a Vanquish HSS T3 column (1.8 µm, 100 × 2.1 mm) was operated with 0.1% formic acid in water (solvent A) and 0.1% formic acid in acetonitrile (solvent B) as eluents with a total flow of 300 µL min^-1^ at 40°C. 10 µL of the sample were injected, and the following elution program was applied: 1 min 95% A/5% B, followed by a linear gradient down to 2% A/98% B within 18 min, followed by flushing to the starting conditions within 3 min. The mass spectrometer was equipped with a heated electrospray ionization source (HESI) operating with the following parameters: 2.5 kV spray voltage, sheath gas flow of 50 a.u., the auxiliary gas flow of 10 a.u., 280°C capillary temperature, and 250°C probe heater temperature. The samples were analyzed in both, positive and negative ionization modes, and the total ion chromatograms (TIC) were recorded in the *m/z* 50 – 750 range, with a resolution of 140,000 at *m/z* 200. The mass axis calibration was performed using a 2×10^-3^ mol L^-1^ solution of sodium acetate.

## **Table S3.** Type of analysis, retention times (RT, min), molar masses, and characteristic fragments.

| Analyte | Type of Analysis | Retention time, min | Molar  Mass, g mol^-1^ | Characteristic  fragment ion(s) |
| --- | --- | --- | --- | --- |
| PENTOL | GC-MS (alcohol) | 7.5 | 86.13 | *m/z* 57 |
| 1-penten-3-one | GC-MS  (oxime dvt. carbonyl) | 11.35 | 84.12 | *m/z* 279 |
| propionaldehyde | GC-MS  (oxime dvt. carbonyl) | 9.46 | 58.08 | *m/z* 236, *m/z* 253 |
| 2,2,6,6-cyclo-hexanone-d_4_ (Int. std.) | GC-MS  (oxime dvt. carbonyl) | 14.025 | 102.18 | *m/z* 297 |
| HEXOL | GC-MS (alcohol) | 11.70 | 100.16 |  |
| HEXOL | LC-MS (PDA detector) | 8.10 | 100.16 | Abs. max. 218 nm |
| butyraldehyde |  | 10.691 | 72.11 | *m/z* 239, *m/z* 267 |
| HEXAL | GC-MS  (oxime dvt. carbonyl) | 13.767, 13.925 | 98.14 | *m/z* 293 |
| HEXAL | LC-MS (PDA detector) | 9.16 | 98.14 | Abs. max. 222 nm |
| HEXAL | LC-MS (MS detector) | 9.29, 9.31 | 98.14 | *m/z* 99.080441 [M+H]^+^ |
| C_6_H_10_O_2_ | LC-MS | 6.6 | 114.14 | *m/z* 113.06080 [M-H]^-^ |

### *5.1. PHOTOOXIDATION OF PENTOL IN PRESENCE OF ^•^OH*


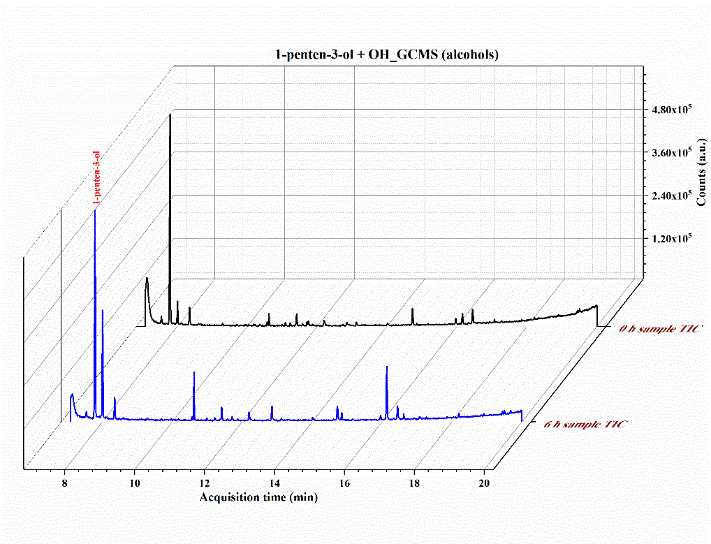


***a)*** ***1-penten-3-ol + OH_GCMS (alcohols)***


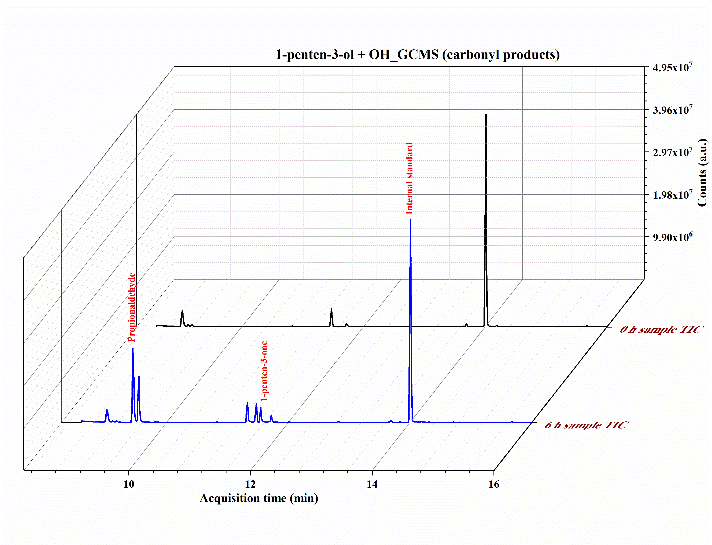


***b) 1-penten-3-ol + OH_GCMS (carbonyls)***

***6 h sample TIC***

***0 h sample TIC***

***1-penten-3-ol***

***Acquisition time (min)***

***Propanal***

***1-penten-3-one***

***Int. standard***

***6 h sample TIC***

***0 h sample TIC***

***Acquisition time (min)***

***Counts (a.u.)***

***Counts (a.u.)***

#### **Fig. S2.** cGC-MS Total Ion Chromatogram (TIC) obtained for the photo-oxidation reaction samples of 1-penten-3-ol (PENTOL) with ^•^OH radicals at time: t = 0 h (black), and t = 6 h (blue). **a)** c-GC-MS TIC for PENTOL (RT = 7.5 min); **b)** c-GC-MS TIC of the carbonyl products. (See Table S3).


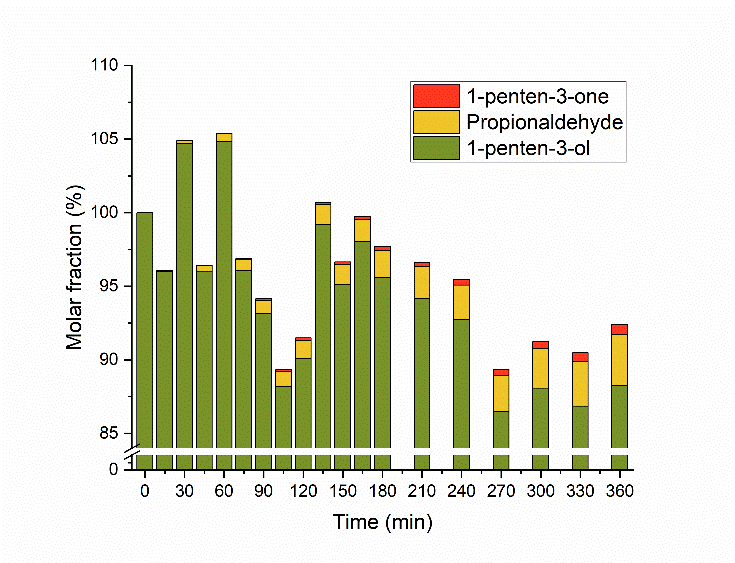


**a)**

**b)**


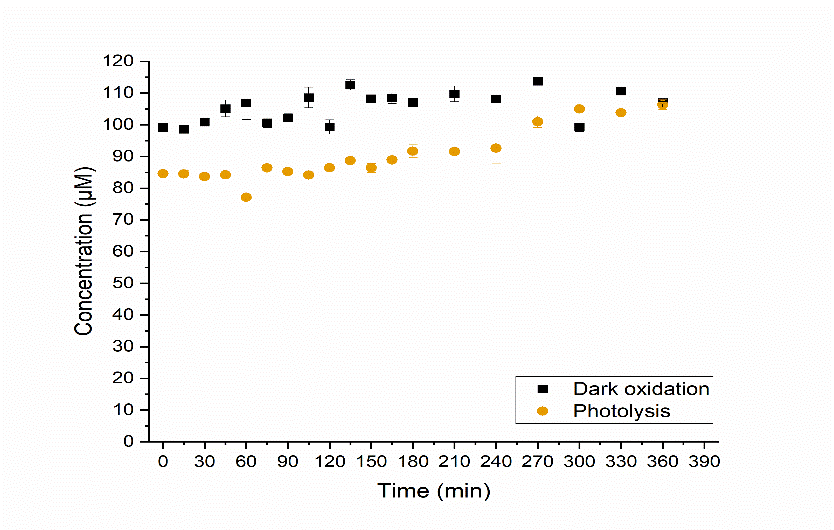


#### **Fig. S3.** a) Molar percent (%) of 1-penten-3-ol (PENTOL) and its ^•^OH-driven oxidation products in aqueous phase; b) Concentration-time profile of PENTOL photolysis (n=1) and dark oxidation (n=1) as control experiments.

### *5.2 PHOTOXIDATION OF HEXOL IN PRESENCE OF ^•^OH*


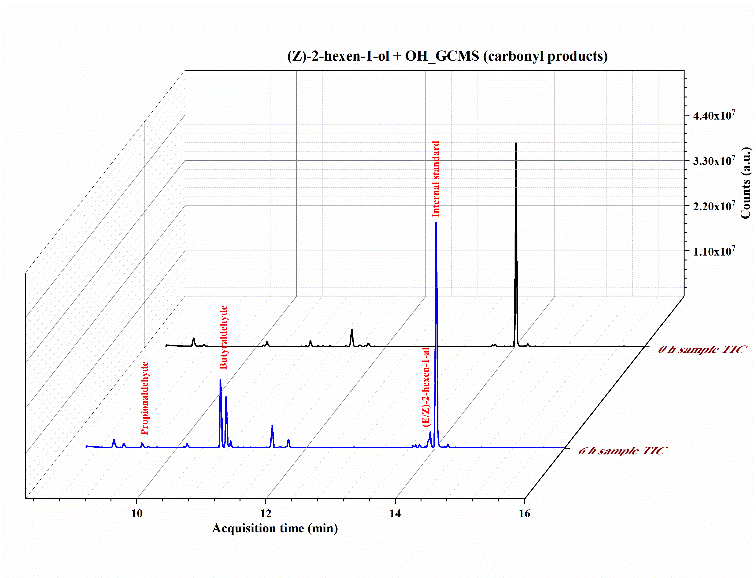

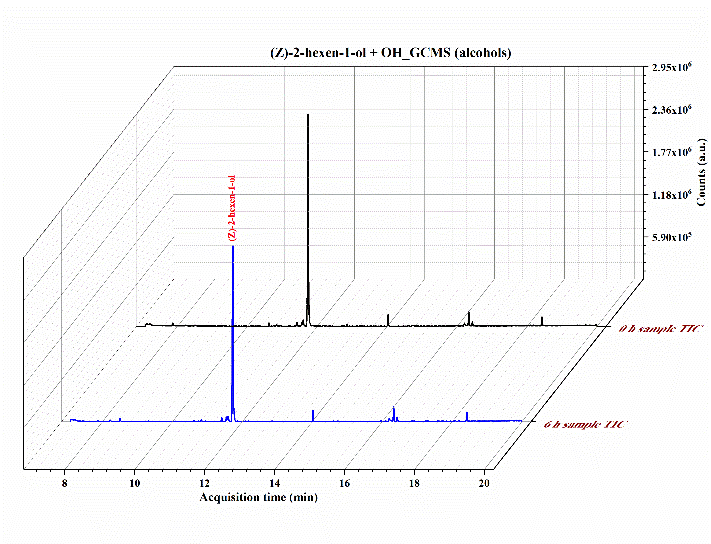


**a) (*Z*)-2-hexen-1-ol + OH_GCMS (alcohols)**

**b) (*Z*)-2-hexen-1-ol + OH_GCMS (carbonyls)**

***Acquisition time (min)***

***Acquisition time (min)***

***(Z)-2-hexen-1-ol***

***propanal***

***butanal***

***Int. standard***

***HEXAL***

***6 h sample TIC***

***0 h sample TIC***

***Counts (a.u.)***

***Counts (a.u.)***

#### **Fig. S4.** cGC-MS Total Ion Chromatogram (TIC) obtained for the photooxidation reaction samples of (Z)-2-hexen-1-ol (HEXOL) with ^•^OH radicals at time: t = 0 h (black), and t = 6 h (blue). **a)** c-GC-MS TIC for HEXOL (RT = 11.7 min); **b)** cGC-MS TIC of carbonyl products. (See Table S3).


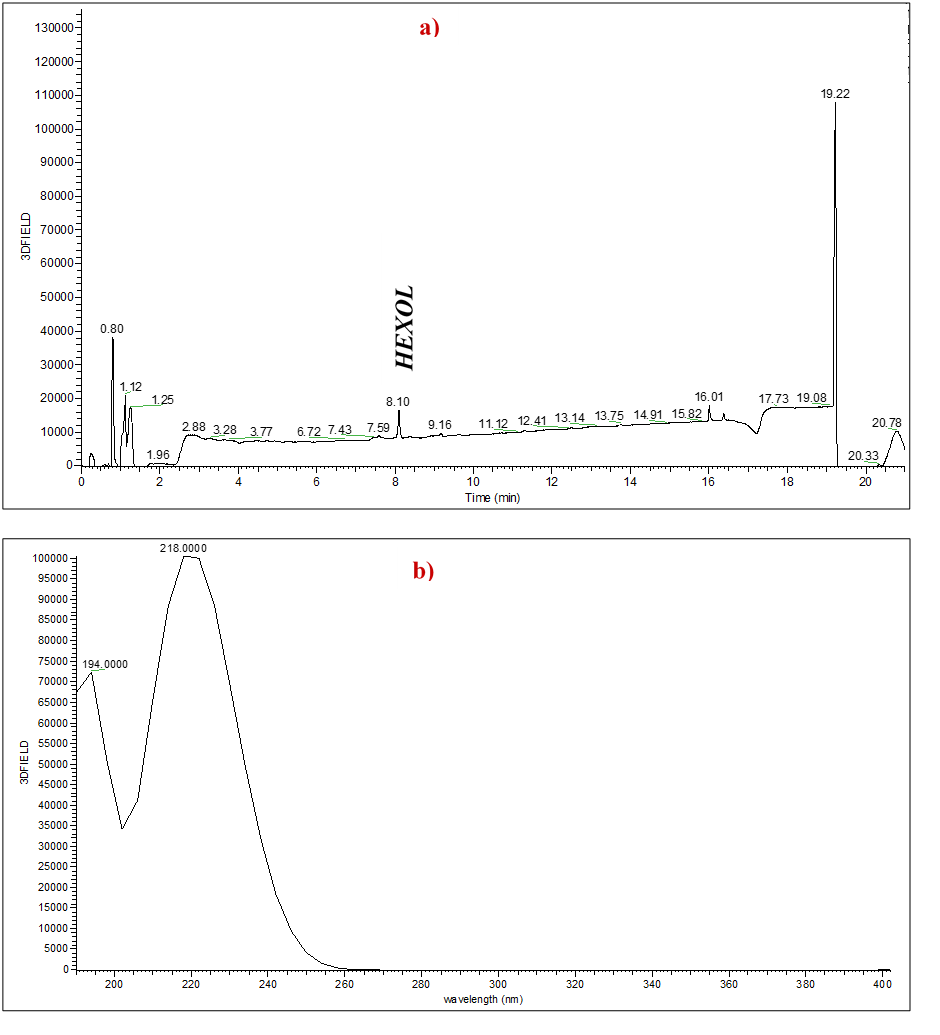
**Fig. S5.** HPLC-PDA/UV total scan of reaction sample, from the photooxidation experiment of HEXOL with ^•^OH radicals at time, t = 6 h. ***a)*** Observed peak on LC chromatogram for HEXOL with a RT 8.1 min, confirmed using an authentic standard. ***b)*** PDA absorbance spectra (190 - 400 nm) of HEXOL, with a characteristic absorbance at 218 nm (See Table S3).


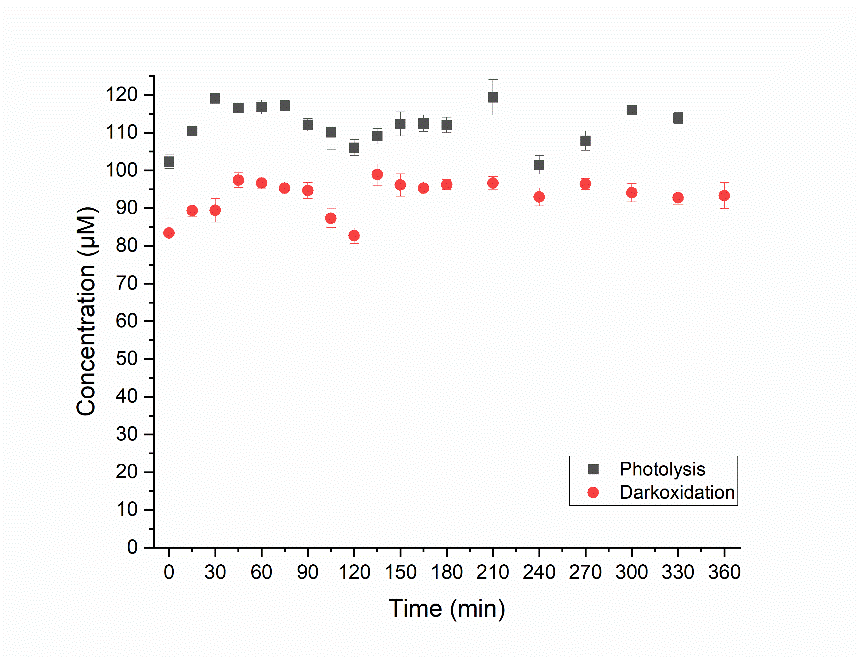

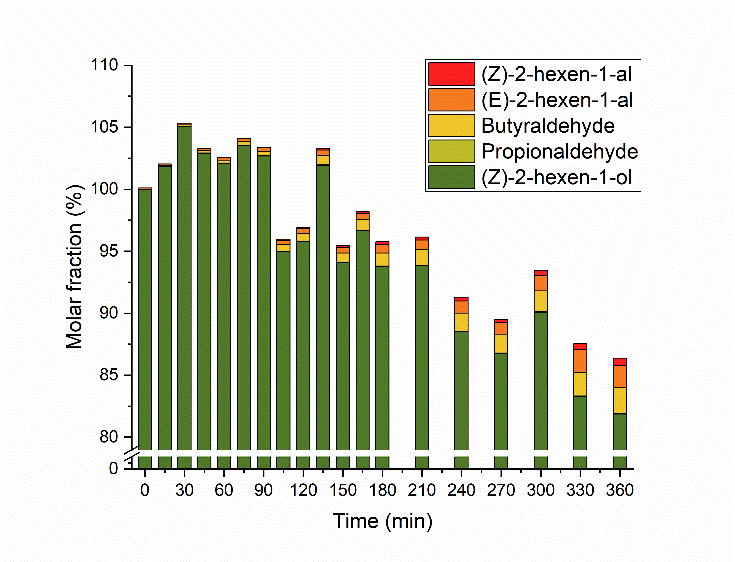


**a)**

**b)**

**Fig. S6.** *a)* Molar fraction (%) of HEXOL and its ^•^OH-driven oxidation products in aqueous phase; *b)* Concentration-time profile of HEXOL photolysis (n=1) and dark oxidation (n=1) as control experiments.

**a)**

**b)**

**C_6_H_10_O_2_**

#### **Fig. S7.** HPLC-ESI/MS Extracted Ion Chromatogram (EIC) acquired for the m/z 113.06080 [M−H]^−^ ion observed in the photooxidation samples containing 0.1 mM HEXOL with 5.0 mM H_2_O_2_ exposed to the UV light in a negative ion mode at: **a)** t = 0 h; **b)** t = 6 h; RT 6.6 min.


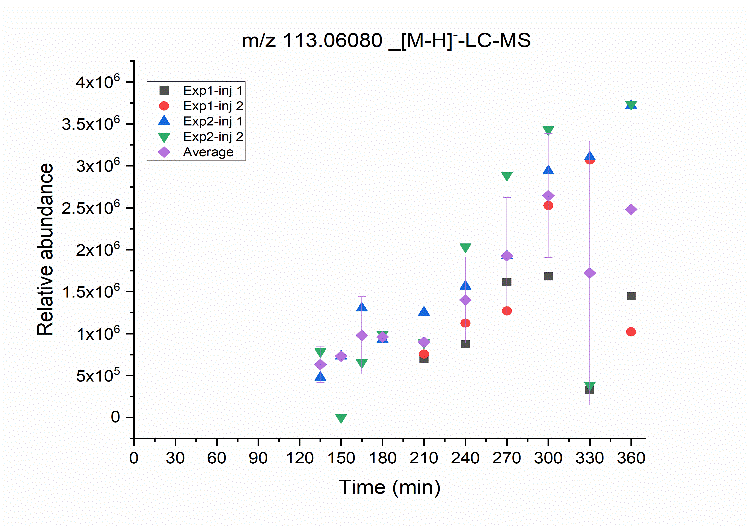


**b)**

**a)**

#### **Fig. S8. a)** HPLC-ESI/MS Mass spectra of m/z 113.06080 [M−H]^−^, corresponding to C_6_H_10_O_2_, RT 6.6 min observed during the photooxidation of (Z)-2-hexen-1-ol with ^•^OH radicals at 6 h. (corresponding chromatogram Figure S7b); **b)** Signal intensity-time profile of m/z 113.0608 during the ^•^OH driven photooxidation of HEXOL (0 – 360 min).

### ***5.3*** *PHOTOOXIDATION OF HEXAL IN PRESENCE OF ^•^OH*

***1) propanal***

***2) butanal***

***3) bdl, m/z 279***

***4) (/Z)-2-hexen-1-al***

***5) (E)-2-hexen-1-al***

***6) Int. standard***


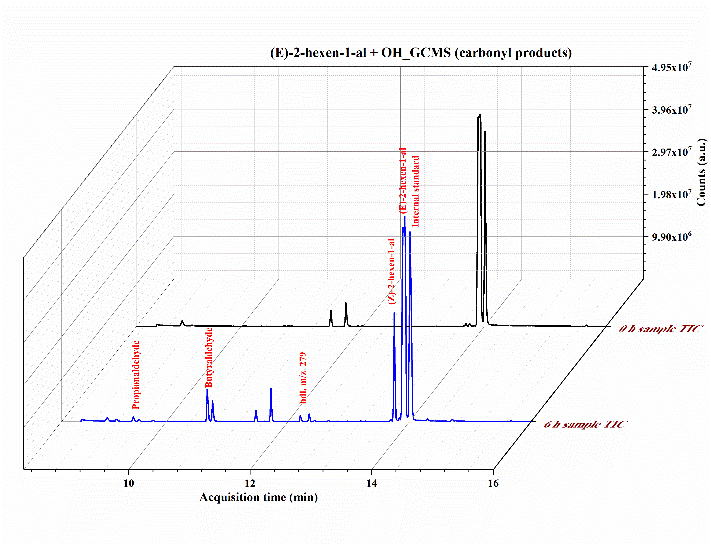


**a) (*E*)-2-hexen-1-al + OH_GCMS (carbonyls)**

**b)**

***1)***

***2)***

***3)***

***4)***

***5)***

***6)***

***Acquisition time (min)***

***Counts (a.u.)***


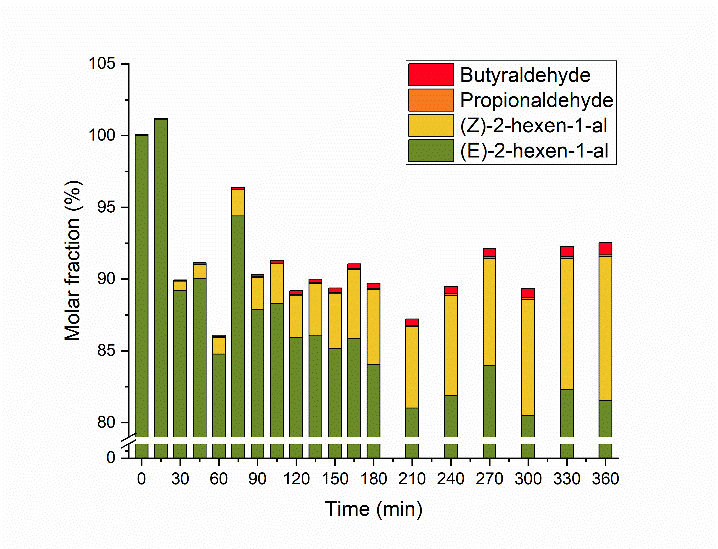


#### **Fig. S9.** **a)** cGC-MS Total Ion Chromatogram (TIC) obtained for the photooxidation reaction samples of (E)-2-hexen-1-al (E-HEXAL) with ^•^OH radicals at time: t = 0 h (black), and t = 6 h (blue) (See Table S3); **b)** Molar fraction (%) of HEXAL and its ^•^OH-driven oxidation products in aqueous phase.


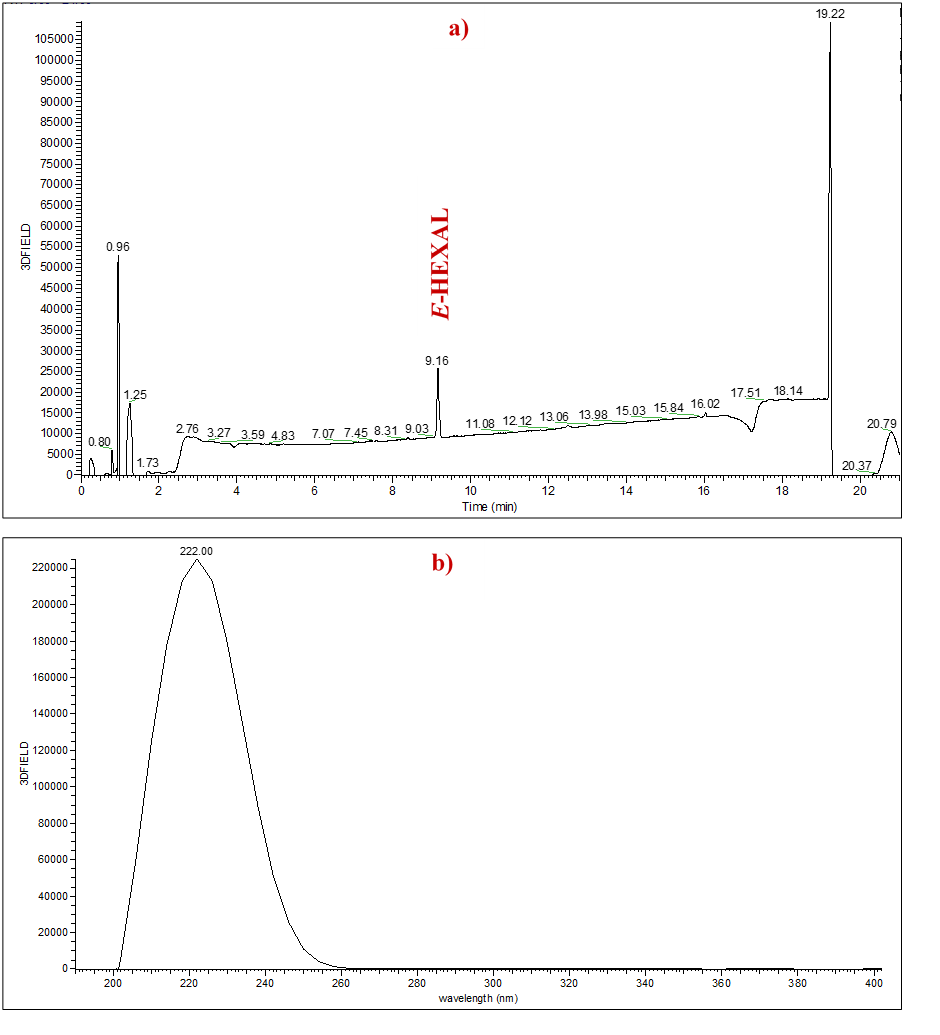
**Fig. S10.** HPLC-PDA/UV data from the photooxidation experiment of E-HEXAL with ^•^OH radicals at time, t = 6 h sample. **a)** Observed peak in the LC chromatogram for E-HEXAL with the RT 9.16 min, confirmed using an authentic standard. **b)** PDA absorbance spectra (190 – 400 nm) of E-HEXAL with a characteristic absorbance maximum at 222 nm (See Table S3).

***E*-HEXAL**

**a)**

**b)**


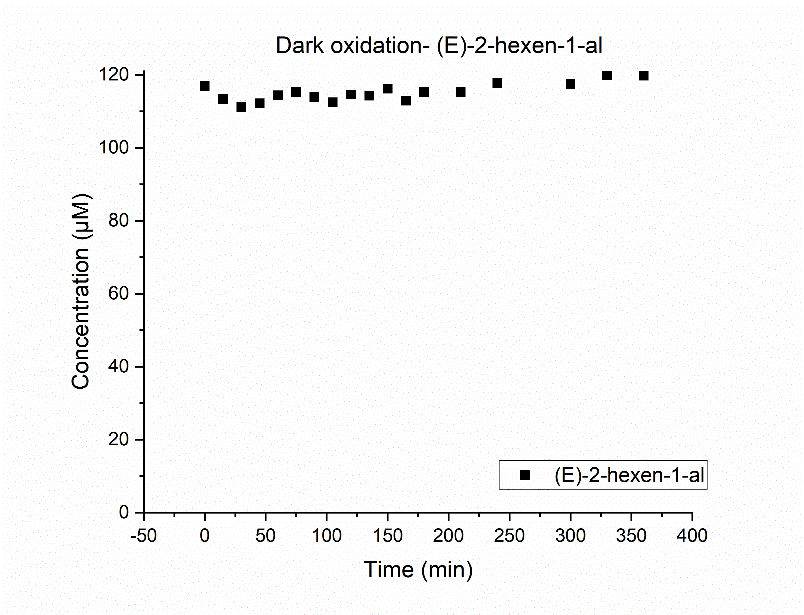

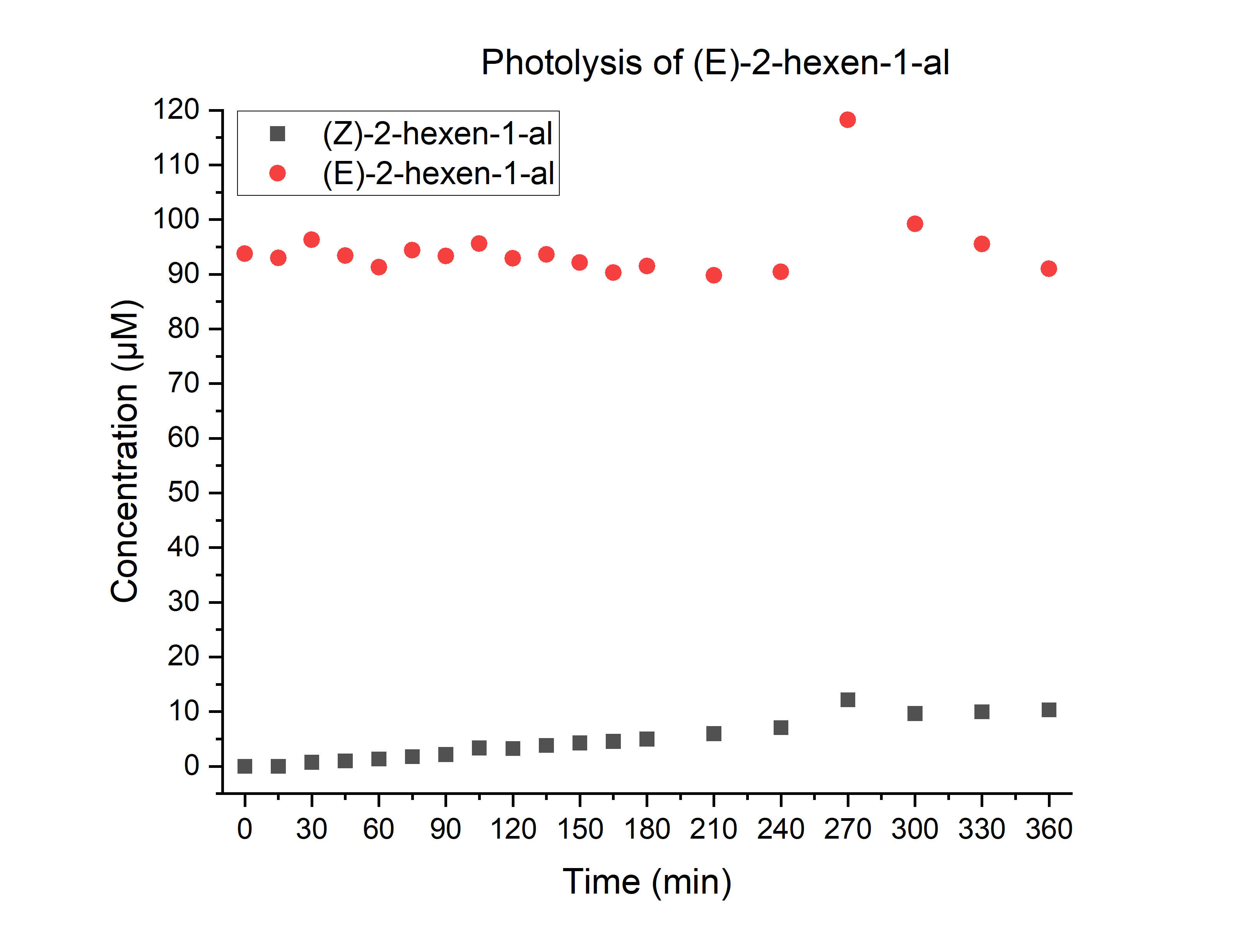


#### **Fig. S11.** Concentration-time profile of reactant E-HEXAL during: **a)** dark-oxidation experiments (n=1); **b)** photolysis (n=1) as control experiments.

**a)**

**b)**

#### **Fig. S12.** HPLC-ESI/MS EIC data of m/z 113.06080 [M−H]^−^ product observed in photooxidation experiments with 0.1 mM (E)-2-hexen-1-al and 5.0 mM H_2_O_2_ exposed to the UV light in a negative ion mode. **a)** at t = 0 h; **b)** at t = 6 h, RT 6.6 min (See Table S3).

**a)**

**b)**


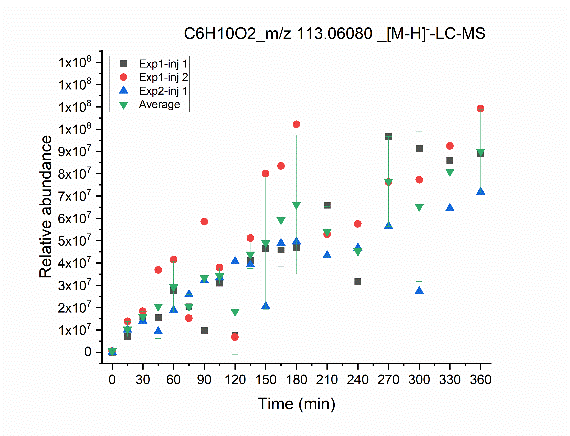


#### **Fig. S13.** **a)** HPLC-ESI/MS mass spectrum of m/z 113.06080 [M−H]^−^ product, corresponding to a C_6_H_10_O_2_ formula at the RT 6.6 min observed during the photooxidation of (E)-2-hexen-1-al with ^•^OH radicals over 6 h. **b)** Signal intensity-time profile of m/z 113.0608 during the ^•^OH radicals-driven photooxidation of HEXAL (0 – 360 min).

# **6. DENSITY FUNCTIONAL THEORY**

**a)**


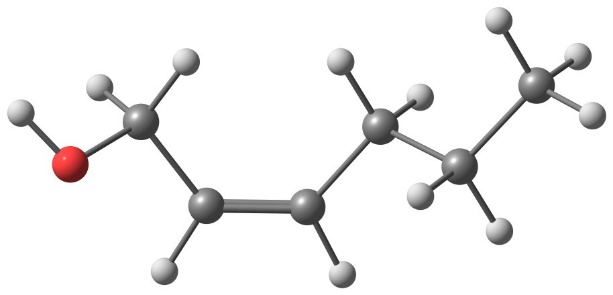

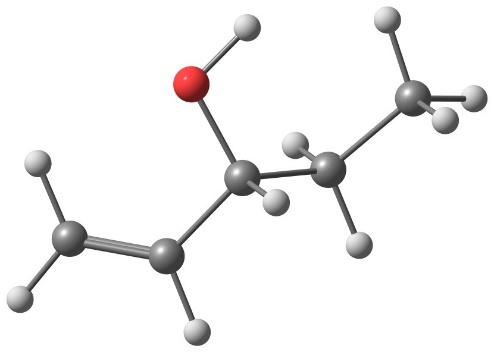


**1**

**2**

**3**

**4**

**5**


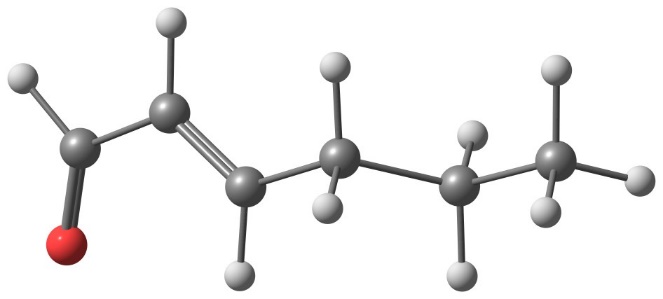


**1**

**2**

**3**

**4**

**5**

**6**

**1**

**2**

**3**

**4**

**5**

**6**

**b)**

**c)**

## **Fig. S14.** Vacuum optimized geometries of green leaf volatile compounds (GLVs) at B3LYP functional and def2-TZVP basis set: ***a)*** 1-penten-3-ol; ***b)*** (*Z*)-2-hexen-1-ol; ***c)*** (*E*)-2-hexen-1-ol (with atom numbers) where, **C**: **grey**, **H**: white, and **O**: red. (C=C 1.33 Å , C-C 1.50 Å ,C-H 1.50 Å, C-O 1.42 Å , O-H 0.96 Å)

## **Table S4.** XYZ coordinates of vacuum optimized structures of selected GLVs

**1-penten-3-ol, C_5_H_10_O**

C 0.397115000 1.209445000 -0.077502000

C -0.237127000 1.420045000 1.066822000

H 0.983220000 1.989428000 -0.544132000

H 0.342882000 0.251290000 -0.578177000

H -0.823628000 0.625847000 1.519824000

C -0.216055000 2.697551000 1.849736000

C 0.308400000 2.463068000 3.273346000

H -0.294535000 1.674616000 3.733562000

H 1.330509000 2.082590000 3.198550000

C 0.269471000 3.712937000 4.148441000

H -0.742493000 4.123122000 4.209831000

H 0.597931000 3.490195000 5.164768000

H 0.931214000 4.494177000 3.766151000

O 0.568858000 3.657733000 1.148914000

H 0.484922000 4.503901000 1.598658000

H -1.253370000 3.062785000 1.928178000

**(*Z*)-2-hexen-1-ol, C_6_H_12_O**

C 0.508859000 -0.851735000 -2.837206000

H 0.975582000 -0.738384000 -1.861418000

C 0.192429000 -2.085997000 -3.220791000

H 0.408461000 -2.913032000 -2.551289000

C -0.443111000 -2.498242000 -4.507666000

H -0.751877000 -1.630888000 -5.098445000

H 0.284050000 -3.066201000 -5.104832000

C -0.776220000 2.749244000 -3.492900000

H -1.257263000 2.610780000 -4.464452000

H -1.398165000 3.429769000 -2.908302000

C -0.588552000 1.414306000 -2.778124000

H -1.561192000 0.949936000 -2.592419000

H -0.136606000 1.581626000 -1.795258000

H 0.184131000 3.241343000 -3.667103000

C 0.283004000 0.433328000 -3.573428000

H 1.252065000 0.908124000 -3.770851000

H -0.173123000 0.254625000 -4.549037000

O -1.571146000 -3.324356000 -4.197477000

H -1.889348000 -3.724846000 -5.013329000

**(*E*)-2-hexen-1-al, C_6_H_10_O**

C 0.347932000 0.694641000 -2.310386000

H -0.163834000 1.332869000 -1.561139000

C 0.363898000 -0.743740000 -1.989449000

H -0.104579000 -1.041306000 -1.056214000

C 0.922111000 -1.651146000 -2.795097000

H 1.375473000 -1.291383000 -3.715904000

C 1.000468000 -3.114019000 -2.527588000

H 0.478782000 -3.650208000 -3.330147000

H 0.483923000 -3.355421000 -1.594604000

C 2.449675000 -3.622539000 -2.468738000

H 2.983955000 -3.080532000 -1.683522000

H 2.953525000 -3.378620000 -3.408667000

C 2.530253000 -5.124089000 -2.210472000

H 2.023041000 -5.687617000 -2.997339000

H 3.567204000 -5.462264000 -2.172781000

H 2.059879000 -5.385032000 -1.259391000

O 0.840888000 1.195739000 -3.296256000

For XYZ coordinates of the vacuum optimized structures of all other intermediates investigated can be downloaded from the ICM based repository website for the dataset

https://repod.icm.edu.pl/dataset.xhtml?persistentId=doi%3A10.18150%2F1J2I3C

1. 1-penten-3-ol with ^•^OH


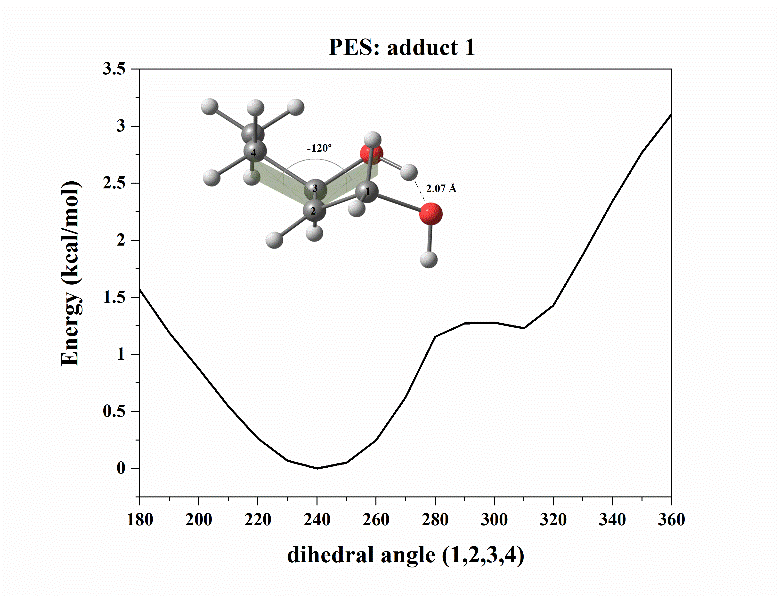

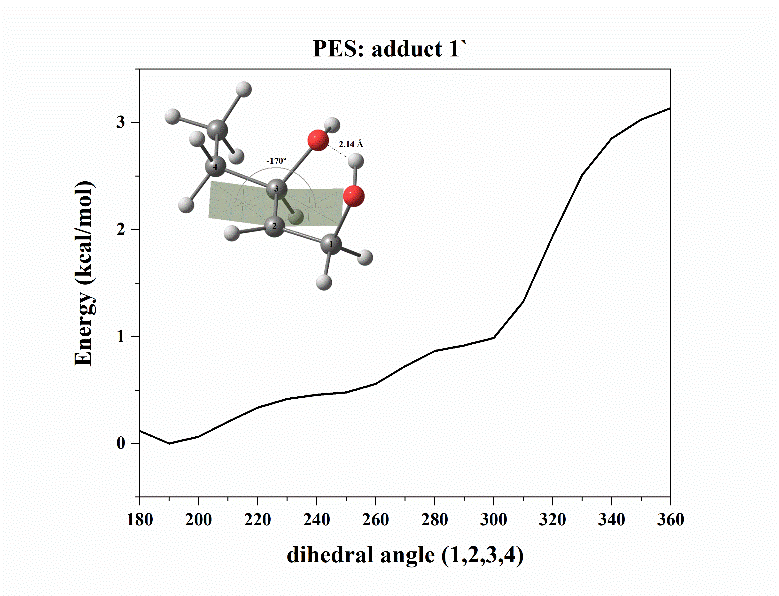

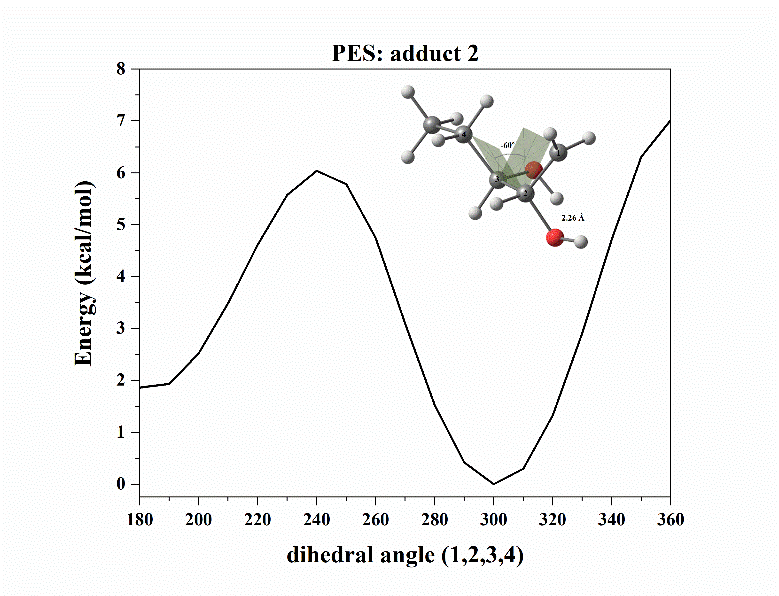

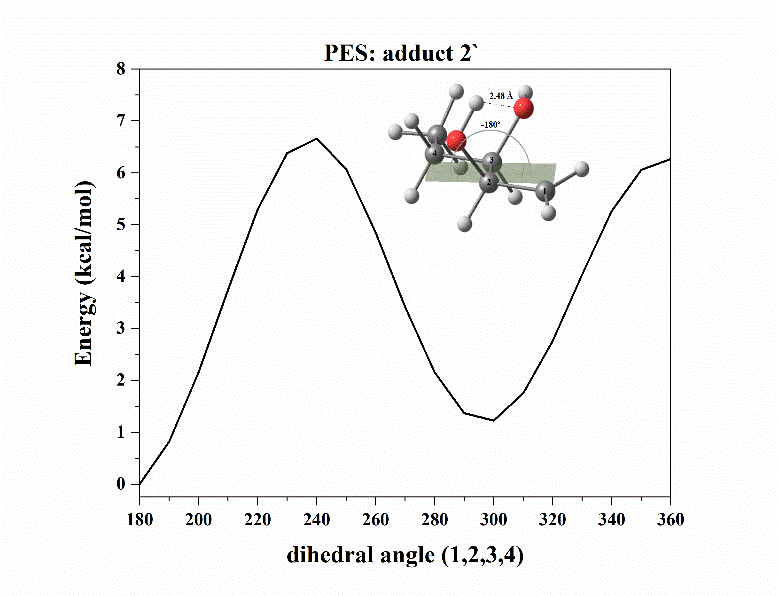


## **Fig. S15.** Potential energy scans (PES) for the dihedral angle between C (1, 2, 3, 4) for the adduct 1, 1`, 2, and 2` resulting from the addition of ^•^OH at C1 and C2 of 1-penten-3-ol.


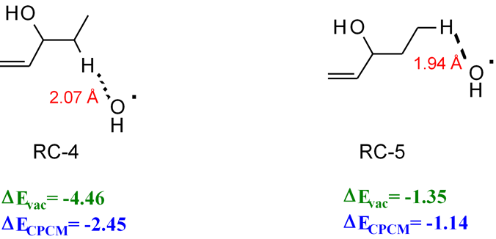


## **Fig. S16.** Other possible higher energy reaction complexes for a hydrogen-abstraction pathway of reaction between 1-penten-3-ol and ^•^OH radicals and their respective relative energy, ∆E shown in vacuum and aqueous phase.

1. (*Z*)-2-hexen-1-ol with ^•^OH







## **Fig. S17.** Potential energy scans (PES) for the dihedral angle between C1, 2, 3, 4 for the adduct 1 and adduct 2 resulting from the addition of ^•^OH at C3 (adduct1) and C2 (adduct2) of (Z)-2-hexen-1-ol.


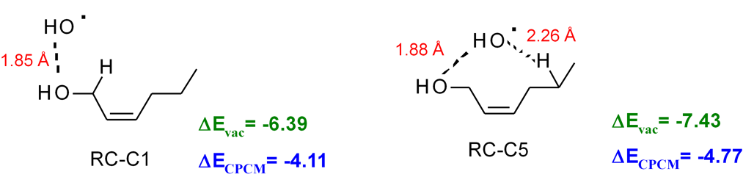


## **Fig. S18.** Other possible higher energy reaction complexes for a hydrogen-abstraction pathway of reaction between (*Z*)-2-hexen-1-ol and ^•^OH radicals and their respective relative energy, ∆E shown in vacuum and aqueous phase.

1. (*E*)-2-hexen-1-al with ^•^OH


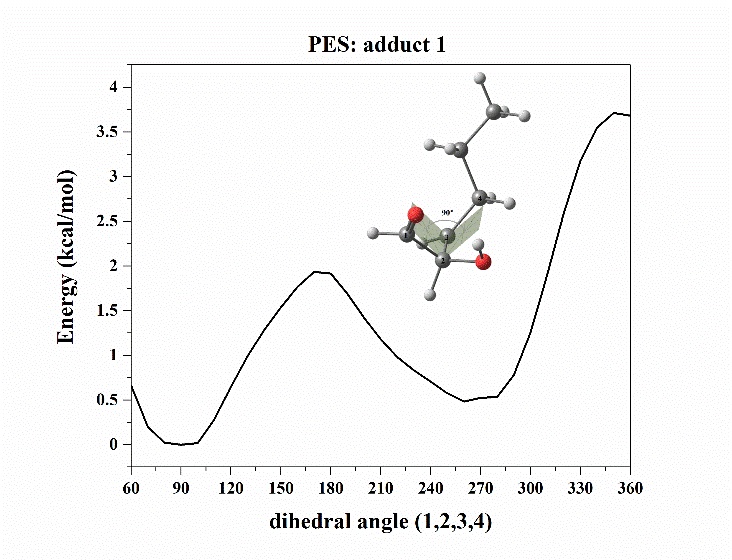

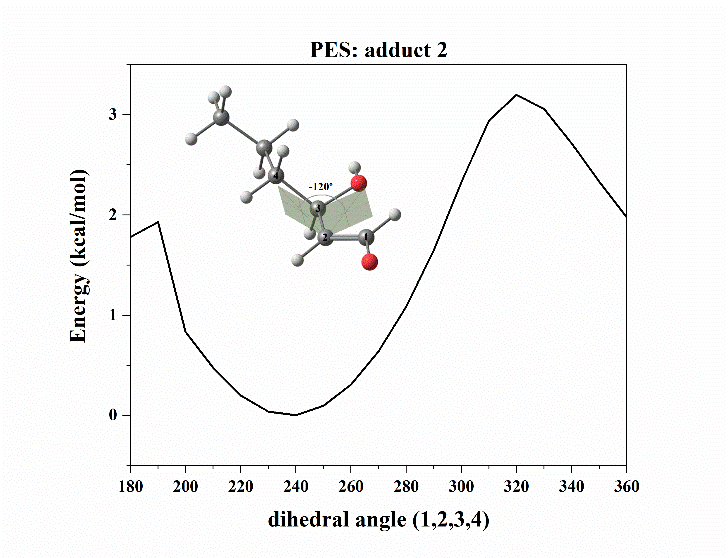


## **Fig. S19.** Potential energy scans (PES) for the dihedral angle between C1, 2, 3, 4 for the adduct 1, and adduct 2 resulting from the addition of ^•^OH at C3 (adduct1) and C2 (adduct2) of *(E*)-2-hexen-1-al.


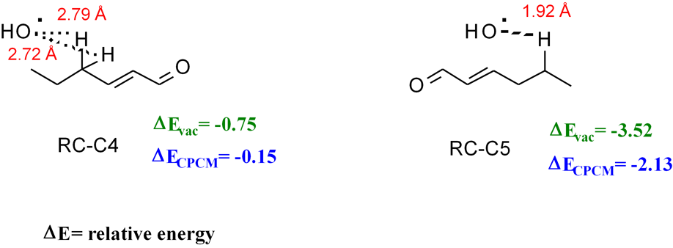


## **Fig. S20.** Other possible higher energy reaction complexes for a hydrogen-abstraction pathway of reaction between (*E*)-2-hexen-1-al and ^•^OH radicals and their respective relative energy, ∆E shown in vacuum and aqueous phase.

# **7. ATMOSPHERIC IMPLICATIONS**

## **Table S5.** Physical properties of GLVs and observed oxidation products at 298 K, experimental and estimated with EPI suite(US_EPA, 2012) and $H_{d}=HRT,$where*, R*, atm L mol^-1^ K^-1^

| Compounds | Molecular  formula | Henry’s Law  Constant | Vapor  pressure | Water  solubility | *H_d_* |
| --- | --- | --- | --- | --- | --- |
|  |  | mol L^-1^ atm^-1^ | atm | mg L^-1^ |  |
| 1-penten-3-ol | C_5_H_10_O | 101.22^a^  43.75^b^ | 1.20×10^-2, c^ | 4.53×10^4^ ^d^  9.01×10^4,^ (Suzuki, 1991) | 2.4×10^3^  1.1×10^3^ |
| (*Z*)-2-hexen-1-ol | C_6_H_12_O | 64.52^a^  133.26^b^ | 1.19×10^-3, c^ | 1.60×10^4^ ^d^ | 1.6×10^3^  3.3×10^3^ |
| (*E*)-2-hexen-1-al | C_6_H_10_O | 10.12^a^  8.63^b^ | 6.21×10^-3, c^ | 5.26×10^3 d^ | 2.5×10^2,f^  2.1×10^2^ |
| 1-penten-3-one | C_5_H_8_O | 28.82^a^  5.18^b^ | 5.03×10^-2, c^ | 2.19×10^4 d^ | 7.0×10^2,f^ |
| Propionaldehyde | C_3_H_6_O | 11.10^a^  1.77^b^  13.62(Buttery et al., 1969) | 4.17×10^-1, c^  4.17×10^-1,^  (Daubert et al., 1985) | 5.108×10^4,^ ^d^  3.06×10^5,^ (Riddick et al., 1986) | 3.3×10^2,f^ |
| Butyraldehyde | C_4_H_8_O | 8.33^a^  2.33^b^  8.70(Buttery et al., 1969) | 1.42×10^-1, c^  1.46×10^-1,^  (Daubert et al., 1985) | 3.018×10^4^ ^d^  7.1×10^4^ ^e^ | 2.1×10^2,f^ |
| Hydroxy-hexenal | C_6_H_10_O_2_ | 2.77×10^5a^  2.65×10^4b^ | 1.03×10^-4, c^ | 3.118×10^5^ ^d^ | 6.8×10^6,f^ |

^a^ Bond estimation method; ^b^ via VP/WSol estimate; ^c^ Mean of Antoine & Grain methods; ^d^ EPI Suite WSKowwin v1.43 Estimate; ^e^ Cited as Union Carbide (1974) by EPA database; ^f^ H_d_ calculated using bond-estimation values and used in calculation of gas-aqueous partitioning of products

## *Oxidation product partitioning between gas and aqueous phases*

Similar to the GLV partitioning calculations by Sarang et al. (2021) we assume that the gas- and aqueous forms of the oxidation products behave equilibria according to Henry’s Law (Equation S4).

$\left[ OxProd \right]_{aq}=H_{d,OxProd}\left[ OxProd \right]_{g}$ (S4)

with dimensionless Henry’s constants, *H_d_* defined by Equations (S5 and S6).

$H_{d}=HRT, if H is in \mathrm{mol}L^{-1}\mathrm{atm}^{-1}$ (S5)

$H_{d}=HRT\rho, if H is in \mathrm{mol}\mathrm{kg}^{-1}\mathrm{atm}^{-1}$ (S6)

Between system of various liquid water content (*ω*), the GLV and its oxidation product (*OxProd*) partition between the phases according to Henry's Law based on Equation S7

$\frac{\left[ OxProd \right]_{g}}{\left[ {OxProd}^{'} \right]_{aq}}=\frac{\left[ OxProd \right]_{g}}{\left[ OxProd \right]_{aq}\omega}=\frac{1}{H_{d}\omega}$ (S7)

where *ω*, m^3^ m^-3^ is the liquid water contents of the atmospheric system; [ ]g and [ ]aq, M are the concentrations in the gas- and aqueous- phases, and *[OxProd’]_aq_* is the oxidation product concentration in the aqueous phase per volume of the gas phase, respectively.





## Fig. S21. Gas-aqueous partitioning of oxidation products of GLV-OH reactions in various atmospheric systems. Y-axis represents GLV or GLV-oxidation products (OxProd)

# **8. REFERENCES**

Buttery, R.G., Ling, L.C., Guadagni, D.G., 1969. Food volatilities- Volatilities of aldehydes ketones and esters in dilute water solution. J. Agric. Food Chem. 17, 385-389.

Christensen, H., Sehested, K., Corfitzen, H., 1982. Reactions of hydroxyl radicals with hydrogen-peroxide at ambient and elevated-temperatures. J. Phys. Chem. 86, 1588-1590.

Daubert, T.E., Danner, R.P., Design Institute for Physical Property, D., American Institute of Chemical, E., 1985. Data compilation tables of properties of pure compounds. Design Institute for Physical Property Data, American Institute of Chemical Engineers, New York, N.Y. (345 E. 47th St., New York 10017).

Elliot, A.J., Buxton, G.V., 1992. Temperature-dependence of the reactions OH + O^2-^ AND OH + HO_2_ in water upto 200-degrees-C. J. Chem. Soc.-Faraday Trans. 88, 2465-2470.

Hansel, A.K., Ehrenhauser, F.S., Richards-Henderson, N.K., Anastasio, C., Valsaraj, K.T., 2015. Aqueous-phase oxidation of green leaf volatiles by hydroxyl radical as a source of SOA: Product identification from methyl jasmonate and methyl salicylate oxidation. Atmos. Environ. 102, 43-51.

Hindmarsh, A.C., 1983. ODEPACK, A systematized collection of ODE solvers. Scientific Computing 1, 55-64.

Hoops, S., Sahle, S., Gauges, R., Lee, C., Pahle, J., Simus, N., Singhal, M., Xu, L., Mendes, P., Kummer, U., 2006. COPASI- A COmplex PAthway SImulator. Bioinformatics 22, 3067-3074.

Otto, T., Schaefer, T., Herrmann, H., 2019. Aqueous-phase oxidation of cis-beta-Isoprene epoxydiol by hydroxyl radicals and its impact on atmospheric Isoprene processing. J. Phys. Chem. A 123, 10599-10608.

Otto, T., Stieger, B., Mettke, P., Herrmann, H., 2017. Tropospheric aqueous-phase oxidation of isoprene-derived dihydroxycarbonyl compounds. J. Phys. Chem. A 121, 6460-6470.

Pastina, B., LaVerne, J.A., 2001. Effect of molecular hydrogen on hydrogen peroxide in water radiolysis. J. Phys. Chem. A 105, 9316-9322.

Petzold, L., 1983. Automatic selection of methods for solving stiff and nonstiff systems of ordinary differential equations. SIAM Journal on Scientific and Statistical Computing 4, 136-148.

Richards-Henderson, N.K., Hansel, A.K., Valsaraj, K.T., Anastasio, C., 2014. Aqueous oxidation of green leaf volatiles by hydroxyl radical as a source of SOA: Kinetics and SOA yields. Atmos. Environ. 95, 105-112.

Riddick, J.A., Bunger, W.B., Sakano, T.K., 1986. Organic solvents: physical properties and methods of purification. Fourth edition. John Wiley and Sons, NY, United States.

Sarang, K., Otto, T., Rudzinski, K., Schaefer, T., Grgic, I., Nestorowicz, K., Herrmann, H., Szmigielski, R., 2021. Reaction kinetics of green leaf volatiles with sulfate, hydroxyl, and nitrate radicals in tropospheric aqueous phase. Environ. Sci. Technol. 55, 13666-13676.

Schaefer, T., Schindelka, J., Hoffmann, D., Herrmann, H., 2012. Laboratory kinetic and mechanistic studies on the OH-initiated oxidation of acetone in aqueous solution. J. Phys. Chem. A 116, 6317-6326.

Suzuki, T., 1991. Development of an automatic estimation system for both the partition-coefficient an aqueous-solubility Journal of Computer-Aided Molecular Design 5, 149-166.

US_EPA, 2012. Estimation Programs Interface Suite™ for Microsoft® Windows, v 4.11. United States Environmental Protection Agency, Washington, DC, USA.
